# Epidemic curves and the profile of patients hospitalized by COVID-19 in a border region

**DOI:** 10.1590/1518-8345.6772.4296

**Published:** 2024-09-23

**Authors:** Merielly Kunkel, Érica Alves Ferreira Gordillo, Laiz Mangini Cicchelero, Franz Porzsolt, Mara Cristina Ripoli Meira, Helder Ferreira, Neide Martins Moreira, Larissa Djanilda Parra da Luz, Nathalia Halax Orfão, Reinaldo Antonio Silva-Sobrinho

**Affiliations:** 1 Universidade Estadual do Oeste do Paraná, Centro de Educação Letras e Saúde, Foz do Iguaçu, PR, Brazil.; 2 Institute of Clinical Economics, Germany.; 3 Secretaria de Saúde de Foz do Iguaçu, Vigilância Epidemiológica, Foz do Iguaçu, PR, Brazil.; 4 Universidade Federal de Rondônia, Porto Velho, RO, Brazil.

**Keywords:** COVID-19, Communicable Disease Control, Hospitalization, Public Health, Border Health, Pandemics

## Abstract

**Objective:**

to describe the epidemic curves and analyze the epidemiological profile of patients hospitalized with COVID-19 in a triple border city.

**Method:**

descriptive-quantitative. The population consisted of COVID-19 cases that required hospitalization, analyzing variables such as: age, gender, race/color, city where they lived, occupation, pregnant woman, institutionalized patient and evolution. Descriptive statistical analysis and analysis of variance and chi-square tests were used.

**Results:**

four epidemic curves were identified in the studied period. Among hospitalized cases, males predominated (55%). Cure was the most frequent outcome in curves 1, 2 and 4, but with no statistical difference (p = 0.2916). Curve 3 showed a higher frequency of deaths (41.70%) in relation to cures (38.77%). The mean ages were significantly different between the curves, with curve 4 having the lowest mean age.

**Conclusion:**

it was concluded that the epidemic curves were influenced by different situations; unvaccinated population, easing of restrictive measures, reopening of the Brazil-Paraguay border, interruption of control actions, crowding of people and circulation of new variants of the disease. Through the epidemiological profile of hospitalized patients, it was concluded that being male, of mixed race/color, aged between 61 and 85 years, and being deprived of freedom were associated with hospitalization and the occurrence of death.

## Highlights

**(1)** The third epidemic curve, in April 2021, presented the highest frequency of deaths.

**(2)** Men, with an average age of 56 years, were the most vulnerable group for the disease.

**(3)** The closing and opening of the border impacted the behavior of the epidemic curve.

**(4)** The start of vaccination in the third curve reduced the number of death cases for the next curve.

**(5)** Unfulfilled social isolation by young people affected the dynamics of the pandemic.

## Introduction

 The pandemic caused by the SARS-CoV-2 coronavirus was considered a global public health crisis. In January 2021, the severe acute respiratory syndrome caused by SARS-CoV-2 resulted in more than 83 million confirmed cases and more than 1.8 million deaths globally ^(Cortinovis; Perico; Remuzzi 2021 )^ . The clinical spectrum of COVID-19 (coronavirus disease 2019) is broad, encompassing asymptomatic infection, fever, fatigue, myalgia, mild upper respiratory tract disease, severe viral pneumonia with a risk of death requiring hospital admission, and in some cases can cause death ^(Wu *et al.* 2020 )^ . 

 Brazil, from February 2020 to October 2022, recorded approximately 34 million cases of COVID-19, with an incidence rate of 16,567 cases/100 thousand inhabitants and a fatality rate of 2.0%. Paraná state, in the same period, recorded around 2.8 million cases, with an incidence of 24,076 cases/100 thousand inhabitants (being the third state in the country in number of cases) and a fatality rate of 0.6% ^(Dias *et al.* 2022 )^ . In turn, the city of Foz de Iguaçu-PR, reported 76,839 cases and 1,331 deaths confirmed by the disease until the first week of October 2022, corresponding to an incidence rate of confirmed cases of 29,786/100 thousand inhabitants and a mortality rate of 513/100 thousand inhabitants ^(Álvaro 2021 )^ . 

 The beginning of the pandemic and its expansion through community transmission corresponds to the initial epidemic peak. Unlike the flu virus, the betacoronavirus has a non-seasonal characteristic, which has spread the disease even during the summer ^(Aquino *et al.* 2020 )^ . At the moment when isolation and social distancing measures were relaxed, an increase in the number of cases and deaths resurfaced and a new peak occurred, characterized by an increase in cases, given the lack of vaccine or medicines, as observed in the period. Thus, each country presented an epidemic peak and curve and these occurred at different times during the years 2020 and 2021 ^(Kissler *et al.* 2020 )^ . 

 Due to the extensive territorial range of Brazil’s borders, which is approximately 16,886 kilometers, divided with ten neighboring countries, which are related through tourist, commercial, economic and health means, it has become essential to monitor the epidemiological situation of COVID-19 cases, and its evolution, and the analysis of the impact of the pandemic in these regions ^(Dias *et al.* 2022 )^ . 

 Considering that the city of Foz do Iguaçu-PR is part of the largest triple border region in Brazil, being among Brazil, Paraguay and Argentina, characterized as the main border of South America in terms of population, daily circulation of people from all continents due to international tourism, commercial relations for the purchase and sale of consumer and durable goods and access to health systems ^(Silva-Sobrinho *et al.* 2021 )^ , it became the international border city in the country with the highest incidence of cases and deaths due to COVID-19, justifying its choice to carry out this study. 

 Furthermore, in this space, the high risk of transmission of COVID-19 was heightened by the high circulation of people of different nationalities and, in response, screening measures and closure of land borders (checkpoints), sea borders (ports) and airlines (airports), isolation of high-risk groups, social distancing, diagnoses and mass testing ^(Nagamine; Ferreira; Kruger 2020^ - ^Minussi *et al.* 2020 )^ . 

 Variations in clinical manifestations are made up of differences in age composition, morbidities, social conditions, as well as discrepancies in culture, social structure and health care between countries and regions. Therefore, it became essential to understand and identify the main clinical and epidemiological characteristics of patients with SARS-CoV-2, in order to recognize the profile most vulnerable to the disease ^(Zhou *et al.* 2020 )^ . 

 It is also noteworthy that the political, health and economic problems worsened by the COVID-19 pandemic have highlighted the need to study the peculiarities of this border region ^(Silva-Sobrinho *et al.* 2021 )^ . The objective of this article is to describe the epidemic curves and analyze the epidemiological profile of patients hospitalized for COVID-19 in a triple border municipality. 

## Method

### Study design

 This is a descriptive-quantitative study, with retrospective data, developed in accordance with the guidelines of the Checklist for Reporting Results of Internet E-Surveys guidelines and Strengthening the Reporting of Observational Studies in Epidemiology (STROBE) ^(Elm *et al.* 2007 )^ . 

### Study setting

 Held in the city of Foz do Iguaçu - PR, located on the triple border of Brazil, Paraguay and Argentina. It has a population of 257,971 inhabitants ^(Instituto Brasileiro de Geografia e Estatística 2021 )^ . 

 During the pandemic period, the city had 589 hospital beds, 71% of which were provided by the Unified Health System (SUS). It is noteworthy that 97.8% of the city’s population was covered by Family Health Teams. It also has 30 Primary Health Units, three Psychosocial Care Centers and three Emergency Care Units. Both SUS and the city’s private services serve the local and regional population, as well as Argentine and Paraguayan visitors and citizens seeking access to the health system ^(Ministério da Saúde (BR) 2024^ - ^Ministério da Saúde (BR) 2021 )^ . 

### Study population

 The population involved in the study represented the totality of patients who were notified and confirmed for COVID-19 in Foz do Iguaçu-PR from March 2020 to December 2021, obtained through notification through the *Notifica* COVID-19 Database. Cases without information on the diagnosis of COVID-19 by RT-PCR (reverse transcriptase reaction followed by polymerase chain reaction) and confirmation of hospitalization were excluded when analyzing the epidemic curves among hospitalized patients. 

### Period

 The data was collected between March 2020 and December 2021 from *Notifica* COVID-19 database, from the City’s Epidemiological Surveillance Department. 

### Variables under study and data analysis

Initially, epidemic curves were identified according to the epidemiological weeks of COVID-19 obtained in the aforementioned period, as well as the prevalence per 100,000 inhabitants of the municipality of Foz do Iguaçu-PR, with the number of cases presented in the form of column graphs. For this purpose, the weekly frequencies of positive cases were used, with the horizontal axis representing time and the vertical axis representing frequencies. The same graph was also created for the number of hospitalizations that occurred during the periods of the epidemic curves.

 The allocation of periods of epidemiological weeks was carried out based on the epidemiological concept of epidemic curve, designated as a graphic representation of the number of cases of a given disease by the date it started, with each valley forming a new epidemic curve, which provides information such as magnitude, time trend and pattern of spread of cases, among others ^(Dwyer; Groves 2007 )^ . 

Subsequently, patients who tested positive for COVID-19 and were admitted to hospital units in Foz do Iguaçu-PR were analyzed, totaling 2927 cases. At this stage, the profile of these patients was characterized based on the following qualitative variables: gender, age group, race/color, country of residence, city, occupation, institutionalized patient, pregnant woman and evolution. These were summarized and compiled into tables using absolute (n) and relative (%) frequencies for each epidemic curve found. The absolute frequencies of these variables were compared between the curves using the chi-square test. When the assumptions for performing the chi-square test were not met, Fisher’s exact test was performed. When such tests pointed to a statistically significant association between the variables, a standardized and adjusted residual analysis was also carried out, such that residuals above 1.96 indicated a positive association between the variables and those lower than -1.96 indicated an association negative. For all tests, a significance level of 0.05 was adopted. Such analyzes were carried out using the XlStat Version 2014 program.

### Ethical aspects

 Considering Resolution Number 466 of December 12 ^th^ , 2012 of the National Health Council and other guidelines and regulatory standards regarding research involving human beings, the research project was approved by the Ethics Committee for Research Involving Human Beings of the Universidade Estadual do Oeste do Paraná – UNIOESTE under number 4,894,155. 

## Results

### Identification of epidemic curves according to epidemiological weeks of highest prevalence

**Figure 1.**
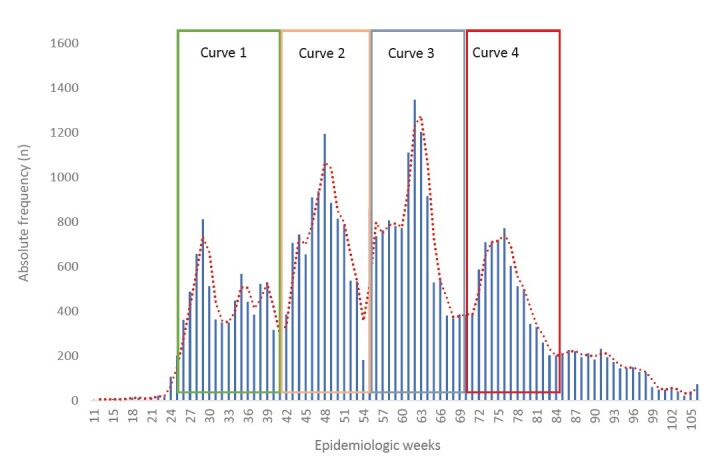
Absolute frequency of positive cases of COVID-19, by epidemiological weeks, from March 2020 to December 2021 in Foz do Iguaçu - PR, Brazil

 As shown in Figure 1 , when evaluating the period from March 2020 to December 2021, 4 epidemic curves were obtained, which covered the following epidemiological weeks: 

 Epidemic Curve 1 - occurred from 05/31/2020 to 10/10/2020, corresponding to the 23 ^rd^ to 41 ^st^ epidemiological week and 7682 people were diagnosed with COVID-19. 

Epidemic Curve 2 - occurred in the period from 10/11/2020 to 01/02/2021, corresponding to the 42nd to 53rd epidemiological week and 9249 people were diagnosed with COVID-19.

 Epidemic Curve 3 - occurred in the period from 01/03/2021 to 04/17/2021, corresponding to the 1 ^st^ to 15 ^th^ epidemiological week and 11,478 people were diagnosed with COVID-19. 

 Epidemic Curve 4 – occurred in the period from 04/18/2021 to 08/07/2021, corresponding to the 16 ^th^ to the 31 ^st^ epidemiological week and 7408 people were diagnosed with COVID-19. 

 Observing the ascending lines of the number of diagnosed cases of COVID-19, there were peaks in weeks 29 and 48 of 2020, and weeks 9 and 22 of 2021 (in the graph identified as weeks 62 and 75). Epidemiological weeks 8 to 11 of 2021 (weeks 61 to 64 of the study), in the third epidemic curve, presented the highest absolute frequencies of positive cases for COVID-19 (n = 1110, 430 cases/100,000 inhabitants; n = 1345, 521 cases/100,000 inhabitants; n = 1201, 465 cases/100,000 inhabitants and n = 914, 354 cases/100,000 inhabitants) ( Figure 1 ). 

### Profile of hospitalized patients in each epidemic curve

**Figure 2.**
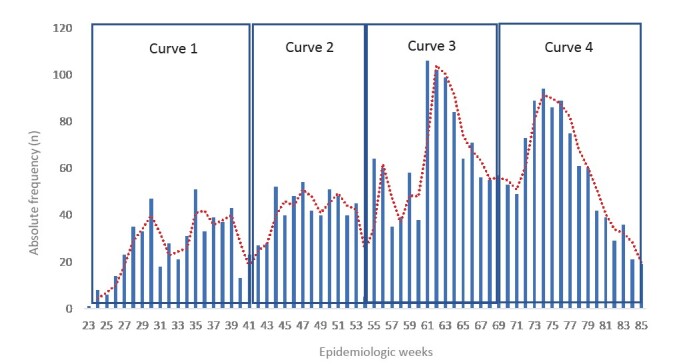
Absolute frequency of hospitalizations due to COVID-19, by epidemiological weeks, in the period of the 2020 and 2021 epidemic curves in Foz do Iguaçu - PR, Brazil

 In total, 504 (6.56% of those diagnosed) patients were hospitalized with COVID-19 during the first epidemic curve, 520 (5.62% of those diagnosed) patients in the second epidemic curve, 988 (8.61% of those diagnosed) patients in the third epidemic curve and 915 (12.35% of those diagnosed) during the fourth epidemic curve. It was found that the highest number of hospitalizations occurred during curves 3 and 4 of the pandemic ( Figure 2 ). 

 It was possible to verify that the majority of hospitalized patients with COVID-19 were male, aged between 46 and 85 years old, white, who lived in their own city and country, had occupations not linked to the area of public safety and health and evolved for healing. A small portion of the cases that required hospitalization were institutionalized or pregnant ( Table 1 ). 

 Among the variables associated with curve 1, age ranged from 0 to 15 years and 61 to 85 years, white and yellow, living in Foz do Iguaçu, Brazil and cases that were cured. Curve 2 was associated with an age of 61 to 85 years, living in Brazil in long-term institutions for the elderly and recovery clinics and progressing to cure. Curve 3 was associated with people aged 61 to 85 years old, of mixed race/color, living in other cities, not in Foz do Iguaçu, and resulting in death. Curve 4 was associated with people aged between 16 and 60 years, mixed race/color, living in other countries and cure outcome ( Table 1 ). 

**Table 1.** Absolute (n) and relative (%) frequencies of qualitative variables of hospitalized patients with COVID-19, for each epidemic curve. Foz do Iguaçu, PR, Brazil, 2020-2021

**Variables**	**Categories**	**Curve 1**	**Curve 2**	**Curve 3**	**Curve 4**	**p-value†**
		n*(%)	n*(%)	n*(%)	n*(%)	
Gender	Male	306(60,7)	291(56,0)	564(57,1)	559(61,1)	0,128 ^[Table-fn tfn09]†^
	Female	198(39,3)	229(44,0)	424(42,9)	356(38,9)	
Age group	0 to 15 years old	17(3,4) ^‡^	10(1,9)	25(2,5)	10(1,1) ^§^	<0,001 ^[Table-fn tfn09]†^
	16 to 30 years old	24(4,8)	29(5,6)	45(4,6)	68(7,4) ^‡^	
	31 to 45 years old	80(15,9)	61(11,7) ^§^	148(14,9) ^§^	226(24,7) ^‡^	
	46 to 60 years old	133(26,4) ^§^	165(31,7)	296(30,0)	338(36,9) ^‡^	
	61 to 85 years old	228(45,2) ^‡^	234(45,0) ^‡^	444(44,9) ^‡^	263(28,7) ^§^	
	86 to 97 years old	22(4,4) ^‡^	21(4,0)	30(3,0)	10(1,1) ^§^	
Race/Color	White	239(57,0) ^‡^	224(51,6)	404(48,7)	382(48,1)	<0,001 ^[Table-fn tfn09]†^
	Brown	89(21,2) ^§^	192(44,2)	394(47,8) ^‡^	379(47,7) ^‡^	
	Yellow	77(18,4) ^‡^	6(1,4) ^-^	10(1,2) ^§^	10(1,3) ^§^	
	Black/Indigenous	14(3,3)	12(2,8)	22(2,7)	23(2,9)	
Country	Brazil	500(99,2) ^‡^	517(99,4) ^‡^	962(97,4)	883(96,5) ^§^	<0,001 ^[Table-fn tfn09]†^
	Others	4(0,8) ^§^	3(0,6) ^§^	26(2,6)	32(3,5) ^‡^	
City	Foz do Iguaçu	504(100) ^‡^	511(98,3)	955(96,7) ^‡^	893(97,6)	<0,001 ^[Table-fn tfn09]†^
	Others	-	9(1,7)	33(3,3) ^‡^	22(2,4)	
Occupation	Others	255(95,5)	352(98,6)	652(99,1)	565(99,8)	- ^||^
	Public safety/Health	12(4,5)	5(1,4)	6(0,9)	1(0,2)	
Institutionalized patients	Prison units	-	1(33,3) ^§^	27(93,1)	10(83,3)	0,036 ^**^
	LCIE ^¶^ and recovery clinics	-	2(66,7) ^‡^	2(6,9)	2(16,7)	
Pregnant	No	80(97,6)	67(94,4)	209(97,7)	268(98,2)	0,304 ^**^
	Yes	2(2,4)	4(5,6)	5(2,3)	5(1,8)	
Evolution	Cure	311(68,4) ^‡^	277(67,2) ^‡^	383(47,8) ^§^	511(65,6) ^‡^	<0,001 ^[Table-fn tfn09]†^
	Death	144(31,6) ^§^	135(32,8) ^§^	419(52,2) ^‡^	268(34,4) ^§^	

* n = Sample, differs since blank, ignored and non-applicable data were not considered in the analysis

† p-value = Chi-square test

‡ Standardized and adjusted residuals above 1.96 that indicated a positive association between the response categories of the variables

§ Residuals below -1.96 that indicated a negative association

|| It was not possible to apply any test for this variable

¶ LCIE = Long-term care institution for the elderly

** p-value of Fisher’s exact test

## Discussion

This study seeks to understand the occurrence of epidemic curves among cases in which people were hospitalized due to COVID-19. The study of epidemic curves is essential for predicting future hospitalization demands, the frequency of emergence of new viral variants and the effectiveness of health interventions, including vaccination.

 The pandemic in Brazil took on increasing proportions between the first and second epidemic curve. In the same period, Brazil and the United States of America were already considered epicenter countries for the disease ^(Gomes *et al.* 2020 )^ . 

 There was also a 161% increase in new cases reported between these two periods ^(Moura *et al.* 2021 )^ , a fact also observed in the study on the collapse of the health system in Manaus, which recorded 56 thousand cases in the first curve and twice as many in the second ^(Barreto *et al.* 2021 )^ . 

 The state of Paraná showed a tendency for accelerate transmission of COVID-19 between the beginning and middle of April 2021. The 9 ^th^ Health Region of Paraná, which involves Foz do Iguaçu-PR, stood out negatively during this period, with higher incidence rate of the disease ^(Banhos *et al.* 2021 )^ . 

 As a measure to mitigate the disease, Foz do Iguaçu adopted the restrictive measures decreed by the state of Paraná and implemented Decree No. 27,994/20, covering prevention measures, aiming to care for the population and keeping the surveillance system on alert ^(Prefeitura Municipal de Foz do Iguaçu 2020a )^ . On the date of publication of the decree, there was still no record of positive infections or suspected cases, with the first positive case, reported on March 18, 2020, classified as an imported case, and the first death from the virus occurred on April 26 ^th^ of the same year. 

 According to Figure 1 , there is a delay between the first confirmed case and the beginning of the first curve, a fact that can be justified by the severe restrictive measures implemented by the city and by Paraguay and Argentina. 

 On the other hand, the first epidemic curve appears at the end of May 2020, a period that coincides with the publication of Decree No. 28,103, which eased restrictive measures, authorizing the opening of commerce although with limits on the number of visitors. This Decree delimited the restrictive measures to neighborhoods of the city with the highest incidence, which were established based on the georeferencing assessment of morbidity and mortality ^(Prefeitura Municipal de Foz do Iguaçu 2020b )^ . 

 The occurrence of the second epidemic curve may be the result of the relaxation of protective isolation measures, as well as the opening of the International Friendship Bridge that connects Brazil to Paraguay and the interruption of actions to control sanitary barriers in all access points to the city ^(Mascarenhas; Klauck 2020 )^ . On this occasion, Ciudad Del Leste, in Paraguay, had an incidence of 1,544 cases per 100,000 inhabitants and a fatality rate of 3.9% and Foz do Iguaçu had an incidence of 3,065 cases per 100,000 inhabitants and a fatality rate of 1.5% ^(Ministério de Salud Publica y Bienestar Social do Paraguay 2022^ - ^Prefeitura Municipal de Foz do Iguaçu [s.d.] )^ . 

 With the opening of the Friendship Bridge, family and work relationships were resumed and, according to the records of the Foz do Iguaçu Health Department, there was a 30% increase in testing for the diagnosis of COVID-19, corresponding to Paraguayans and Brazilians immigrated to the neighboring country, who sought care in the city’s health services ^(Prefeitura Municipal de Foz do Iguaçu [s.d.] )^ . In this study, there was an increase in the number of non-Brazilian residents admitted to hospital in Foz do Iguaçu. It is important to mention that, in general, some border control measures were more effective than others. However, much remains uncertain about this topic ^(Grépin; Aston; Burns 2023 )^ . 

 The third epidemic curve coincided with the period of increased crowding due to the end of the year and carnival festivities. The third wave proved to be the most contagious and lethal, reaching 401 daily cases and a peak of 1,106 active cases ^(Álvaro 2021 )^ . This can be explained in part by the circulation of new variants in Brazil (B.1.1.7, 20B/501Y.V1 or VOC-202012/01) ^(Dejnirattisai *et al.* 2021 )^ , which also reached the city. To control the situation, a new decree was published, number 6983, which determined the suspension of face-to-face classes and non-essential services and activities throughout the state and a curfew from 8 pm to 5 am. This decree was in force until March 8 ^th^ , 2021 ^(Silva-Sobrinho *et al.* 2021 ,Governo do Estado do Paraná (BR) 2021 )^ . 

 A study with data from international borders on all continents showed the effectiveness of the measures introduced to restrict human movement ^(Shiraef *et al.* 2022 )^ , with quarantine being the most effective response to the coronavirus pandemic ^(Grépin; Aston; Burns 2023 )^ . 

 The fourth epidemic curve occurred in a period of easing of restrictive measures ^(Grépin; Aston; Burns 2023 )^ and the circulation of a new variant called Beta (B.1.351 or 501Y.V2), which by February 2021 had been detected in 35 countries, confirming records of this variant in Brazil occurred at the end of April 2021 ^(Dejnirattisai *et al.* 2021 )^ . 

The temporal analysis of the results of this study, the decrees closing and opening borders in Brazil (specifically the border between Foz do Iguaçu and Ciudad del Leste), the easing of isolation and social distancing measures and the emergence of new viral variants led to perception of the relationship between these events and the increase in the number of COVID-19 cases, hospitalizations and the occurrence of deaths.

 The International Friendship Bridge was reopened on October 15 ^th^ , 2020 on the Proclamation of the Republic holiday, this occurred in the 42 ^nd^ epidemiological week and then the other events (mentioned previously) occurred and from then on, according to this research, an increase in the number of cases was also evident in the following epidemiological weeks (45 ^th^ to 48 ^th^ ) in the city of Foz do Iguaçu. 

 However, regarding the closure of international borders, there is information that shows that these may be among the most expensive COVID-19 control measures from an economic point of view ^(Grépin; Aston; Burns 2023 )^ . It is noteworthy that the policies introduced to restrict human movement within a country were more effective in response to the coronavirus pandemic than the closure of international borders ^(Shiraef *et al.* 2022 )^ . 

 Regarding the epidemic curves, the increasing increase in incidence, hospitalization and death due to COVID-19 stands out during the period of the third epidemic curve in the city, during the first four months of 2021, this occurrence was in accordance with the scenario of worsening of the epidemic. pandemic in the international context. During this period, only 13.7% of the Brazilian population had been vaccinated against SARS-CoV-2 ^(Governo do Estado do Paraná (BR) 2021 )^ . 

 Factors such as the course of the disease, the emergence of new variants with greater transmissibility, clandestine gatherings and crowds at the end of the year, added to the growth of the curve, when there were still only non-pharmacological measures to mitigate COVID-19 infection ^(Prefeitura Municipal de Foz do Iguaçu 2021 )^ . 

 The epidemiological profile showed a higher occurrence of hospitalizations among white men. When considering the age groups according to the curves, a higher frequency was noted among hospitalized men aged 61 to 85 years, in the third epidemic curve, aspects in agreement with another study ^(Slavov *et al.* 2021 )^ . 

The analysis of hospitalized cases by age group according to the epidemic curves confirms the high circulation/transmission and number of patients caused by SARS-CoV2 in the locality, as the age group from 0 to 15, 61 to 85 and 86 and over years old, had a higher percentage of hospitalizations during the third epidemic curve (x2 = 132.09; p < 0.0001).

 For patients aged 61 and over, scientific findings corroborate this research, as elderly people who normally have comorbidities such as systemic arterial hypertension, heart disease, diabetes mellitus, chronic respiratory diseases and immunosuppressants are part of a group risk that generally leads to hospitalization and a poor prognosis ^(Moraes *et al.* 2017 )^ . 

 On the other hand, hospitalization and mortality from COVID-19 in hospitalized patients showed a reduction, especially among the elderly, by approximately one third in the fourth epidemic curve. The availability of the vaccine in advance and greater adherence to the second dose could have prevented a significant number of deaths among elderly people in the period from January to April 2021 ^(Jamieson; Rasmussen 2022 )^ . 

 When it comes to the characteristics of those hospitalized according to the epidemic curves, this study was in agreement with findings in South Africa, when comparing the number of curves, (four) periods of occurrence and age of patients during the fourth epidemic curve and diverges regarding presence of a greater number of women in the third epidemic curve ^(Slavov *et al.* 2021 )^ . 

 This study supports the finding that men are more likely to be infected and more frequently require hospitalization for COVID-19 ^(Slavov *et al.* 2021 ,Jamieson; Rasmussen 2022 )^ , potentially due to sex-based immunological differences, gender variations or associated comorbidities, including hypertension, cardiovascular disease, lung disease, patterns and prevalence of smoking and alcohol consumption ^(Silva *et al.* 2021 )^ . Likewise, other findings revealed that 69% of all admissions to hospitals were male, representing 74% of all admissions to the Intensive Care Unit and 77% of all mortality from COVID-19 ^(Naveca *et al.* 2021^ - ^Vahey *et al.* 2021 )^ . 

 In Brazil, small changes occurred in the proportion of elderly and white people infected in the second epidemic curve compared to the first, and a slight increase in hospitalizations was also observed in the second epidemic curve ^(Barreto *et al.* 2021 )^ . However, an increasing number of hospitalizations was observed during 2020 and 2021 in Foz do Iguaçu - PR, not coinciding exactly with the same period in other national scenarios, given that Brazil is a continental, heterogeneous country, with varied population densities and large regional inequities, which determine different times for the beginning and end of each epidemic curve ^(Maslo *et al.* 2022 )^ . 

 The change in the profile of those infected with an increase in the contribution of individuals of mixed race/color in Foz do Iguaçu (from the second curve onwards) may have reflected inequalities in access to health services among whites, mixed race, and black people. Although SARS-CoV-2 affects the population, its impacts may be different due to socioeconomic inequality, structural racism and situations of social exclusion that are part of the Brazilian reality. Those who have more precarious living and working conditions have greater difficulties in accessing essential goods and services, which can then result in differences observable through proxy variables of socioeconomic status, such as race/color ^(Silva-Sobrinho *et al.* 2021 )^ . 

 Regarding the place where they lived, patients hospitalized in Foz do Iguaçu coming from other locations in the state of Paraná and abroad, occurred due to the regionalization of the Unified Health System (since through the Bed Center patients are moved from their municipalities to cities with available beds), the hospitalization of foreigners on tourism and business trips and the access of patients from Paraguay and Argentina. Furthermore, following the opening of the International Friendship Bridge, the number of hospitalizations due to COVID-19 among Brazilians and foreigners from Paraguay increased ^(Prefeitura Municipal de Foz do Iguaçu [s.d.] )^ . 

 In the state of Paraná there was an increase in cases in November 2020, in the 46 ^th^ epidemiological week, in which the average hospital occupancy rate was 89% in the state and 96% in the capital Curitiba ^(Chen; Liu; Guo 2020 )^ . When it comes to the border, the increase in cases was possibly related to the long holiday on November 15 ^th^ , a date when the city of Foz do Iguaçu exceeded expectations for visits since August 2020, with 95% of hotels occupation ^(Grépin; Aston; Burns 2023 )^ . 

 In this study, hospitalization of health professionals was infrequent during the four epidemic curves, contrary to what was observed in a study carried out in Germany and Malaysia, which in the first five months of 2020 alone accounted for 8.9% of the total number of cases. notifications classified as health professionals sick with COVID-19, who needed to be hospitalized ^(Cummings *et al.* 2020 )^ . 

 In the case of institutionalized people, it was observed that during the third epidemic curve, the number of people deprived of their liberty with complications due to COVID-19 and requiring hospitalization increased. The analysis of the institutionalized patients category, regarding the need for hospitalization due to COVID-19, demonstrated that the group of residents in prison institutions were more vulnerable when compared to people outside prisons ^(Fang *et al.* 2020 )^ . 

 The number of pregnant women hospitalized for COVID-19 in this study was low, even during the third epidemic curve. It is known that pregnant women are more likely to be hospitalized in the intensive care unit, require invasive ventilation, require extracorporeal membrane oxygenation and die more frequently than non-pregnant women of reproductive age ^(Nandy *et al.* 2020 )^ . 

 It is noteworthy that the period with the highest morbidity and mortality due to COVID-19 recorded in the municipality was in the third epidemic curve, which corresponded to the period from January to April 2021, presenting 419 deaths, among those who were hospitalized. According to data consolidated by the Coronavirus Panel – Ministry of Health, when Brazil registered the second epidemic curve, the Brazil-Paraguay-Argentina border was experiencing the most critical moment of the third epidemic curve, with a very high percentage of hospitalizations and deaths due to the disease ^(Barreto *et al.* 2021 )^ . 

 Vaccination against SARS-CoV-2 in the municipality began following the guidelines of the 9 ^th^ Health Region of Paraná and health professionals who were on the front line, followed by the elderly sheltered in nursing homes, were the first to receive the vaccine. first dose of the vaccine and during this period the triple border was already experiencing the impacts of the third epidemic curve, which peaked on February 14 ^th^ , 2021, recording more than 1800 new cases in the 8 ^th^ epidemiological week of 2021 ^(Governo do Estado do Paraná (BR) 2021 )^ . 

 In Brazil, with the beginning of summer, infections among young people increased considerably, thus marking the second epidemic curve. Both in Brazil and in other countries, unlike the first moment of the pandemic, positive cases increased among young people during this period. This occurrence can be justified by the fact that a large part of this population believes that they are less susceptible to contamination by COVID-19, as they are not part of risk groups in which there is a predisposition to the disease ^(Goes; Ramos; Ferreira 2020 )^ . 

 Summer, a typical vacation period, and holidays occurring in the third epidemic curve, are other factors that may have contributed to the explosion in the incidence of cases and hospitalizations in Foz do Iguaçu. According to data from the Iguaçu National Park, the number of tourists at Iguaçu Falls in January 2021 was 75 thousand tourists, the highest number recorded since the beginning of the pandemic ^(Grépin; Aston; Burns 2023 )^ . 

 It is noteworthy that during holidays, when people travel and hold meetings, they consequently culminate in crowds and these contribute to the spread of COVID-19 ^(Junior *et al.* 2021 )^ . The spread of the virus on a global scale was favored by the circulation of infected people and objects. However, the health-disease process in a border region has particular characteristics associated with the mobility of people between countries on a daily basis ^(Nienhaus; Hod 2020 )^ . 

Observation of the COVID-19 epidemic curves in the municipality of Foz do Iguaçu shows that the health measures chosen were associated with the morbidity and mortality of the disease. Since, the implementation of restrictive measures before the first epidemic curve was recorded, had an impact on the incidence of the disease and hospitalization and when the municipality adopted relaxation measures there was an increase in the number of cases; subsequently, restrictive measures were intensified, achieving a decrease in the curves after a few weeks.

 Regarding this fact, in the context of a resurgence of SARS-CoV-2, a control strategy through the prohibition of public events and public meetings of more than ten people showed an association with a reduction in cases of around 6% on the 7 ^th^ day, 13% on day 14 and 29% on day 28 ^(Abdalbary *et al.* 2022 )^ . 

 Actions to restrict public adjustments and vaccination coverage in Brazil and border municipalities had exceeded 70% of vaccination coverage for the 1 ^st^ dose and, in the municipality of Foz do Iguaçu, it was 92.5% (first dose) and 86. 3% for the second dose ^(Prefeitura Municipal de Foz do Iguaçu [s.d.] )^ impacted the reduction of hospitalizations in the fourth epidemic curve. 

 In this study, the frequency of hospitalized health professionals was small; a higher frequency was observed during the first epidemic curve. Despite this, the psychosocial and physical repercussions during the pandemic on health, especially for nursing professionals, were severe, given the time of exposure, pace, and complexity of working with people sickened by COVID-19 ^(Ampos *et al.* 2023 )^ . 

It is worth highlighting the existence of incompleteness in some response categories in the database used in the study, this situation may have limited the interpretation of some variables. However, to mitigate bias, 35,817 records of positive cases for COVID-19 were analyzed in the period and only notifications with complete information on the dependent and independent variables were included. Furthermore, the cases considered in the study had confirmation of the diagnosis for SARS-CoV-2 by RT-PCR and hospitalization status.

Identifying the profile of patients hospitalized for COVID-19 in each epidemic curve helps to show the importance of analyzing epidemiological surveillance data with a view to planning health measures. It was possible to identify that although brown people make up 30% of the total population, they still had a similar pattern of illness and death in relation to white people, (63.3% of the total population) it was verified that men and people deprived of freedom are also vulnerable groups, which are potential risk markers for hospitalization and death due to COVID-19. Another contribution to the management of future epidemics is the need to make vaccines available in a timely manner, as in this case, it could have prevented excess illnesses and deaths, as was identified during the third epidemic curve of the disease in this international border location.

## Conclusion

The epidemiological analysis of COVID-19 cases in Foz do Iguaçu that required hospitalization allowed us to conclude that the epidemic curves were influenced by different situations. During the first curve, practically the entire population did not have specific immunity, adding to the aggravating factor of the presence of risk groups and the easing of restrictive measures with the opening of commerce. Preceding the second curve, it marked the reopening of the International Friendship Bridge and the interruption of health barrier control actions. The third epidemic curve was observed in a period after the increase in crowds of people due to the end of the year festivities, carnival and the circulation of new variants in Brazil called B.1.1.7, 20B/501Y.V1 and VOC-202012/01. In turn, the increasing increase in incidence, hospitalization and death due to COVID-19, formed a fourth epidemic curve a period of time after the emergence of another variant of SARS-CoV-2 called Beta (B.1.351 or 501Y .V2).

Regarding the profile of hospitalized patients, it was concluded that the third epidemic curve was the period of greatest transmissibility of the disease, mostly in men, people of mixed race/color, in the age group of 61 to 85 years old and living in a prison unit. These characteristics were associated with hospitalization and death.
