# Supplementary material for: Epidemic curves and the profile of patients hospitalized by COVID-19 in a border region
Source: Rev Lat Am Enfermagem. 2024 Sep 23;32:e4296. doi: 10.1590/1518-8345.6772.4296 (PMC11421522; doi:10.1590/1518-8345.6772.4296)
Supplement: Translation

# Translation: Curvas epidémicas y el perfil de pacientes hospitalizados por COVID-19 en región fronteriza *

**DOI:** 10.1590/1518-8345.6772.4295

**Published:** 2024-09-23

**Authors:** Merielly Kunkel, Érica Alves Ferreira Gordillo, Laiz Mangini Cicchelero, Franz Porzsolt, Mara Cristina Ripoli Meira, Helder Ferreira, Neide Martins Moreira, Larissa Djanilda Parra da Luz, Nathalia Halax Orfão, Reinaldo Antonio Silva-Sobrinho

**Affiliations:** 1 Universidade Estadual do Oeste do Paraná, Centro de Educação Letras e Saúde, Foz do Iguaçu, PR, Brasil.; 2 Institute of Clinical Economics, Alemania.; 3 Secretaria de Saúde de Foz do Iguaçu, Vigilância Epidemiológica, Foz do Iguaçu, PR, Brasil.; 4 Universidade Federal de Rondônia, Porto Velho, RO, Brasil.

**Keywords:** COVID-19, Control de Enfermedades Transmisibles, Hospitalización, Salud Pública, Salud Fronteriza, Pandemias

## Abstract

**Objetivo:**

describir las curvas epidémicas y analizar el perfil epidemiológico de los pacientes hospitalizados por COVID-19 en un municipio de triple frontera.

**Método:**

descriptivo-cuantitativo. La población se configuró de casos de COVID-19 que requirieron hospitalización, analizando variables como: edad, sexo, raza/color, municipio de residencia, ocupación, gestante, paciente institucionalizado y evolución. Se utilizó el análisis estadístico descriptivo y las pruebas de análisis de varianza y chi-cuadrado.

**Resultados:**

se identificaron cuatro curvas epidémicas en el período estudiado. Entre los casos hospitalizados, predominó el sexo masculino (55%). La cura fue el desenlace más frecuente en las curvas 1, 2 y 4, aunque sin diferencia estadística (p = 0,2916). La curva 3 presentó una mayor frecuencia de fallecimientos (41,70%) en relación a la cura (38,77%). El promedio de edad fue significativamente diferente entre las curvas, siendo que la curva 4 presentó el menor promedio de edad.

**Conclusión:**

se concluyó que las curvas epidémicas fueron influenciadas por diferentes situaciones; población no vacunada, flexibilización de las medidas restrictivas, reapertura de la frontera Brasil-Paraguay, interrupción de las acciones de control, aglomeración de personas y circulación de nuevas variantes. Por medio del perfil epidemiológico de los enfermos hospitalizados, se concluyó que ser del sexo masculino, de raza/color pardo, en el rango de edad de 61 a 85 años, estar privado de libertad se asociaron con la hospitalización y ocurrencia de fallecimiento.

## Destacados

**(1)** La tercera curva epidémica, en abril de 2021, presentó la mayor frecuencia de fallecimientos.

**(2)** Hombres, con una edad media de 56 años, fueron el público más vulnerable para la enfermedad.

**(3)** El cierre y apertura de la frontera impactó en el comportamiento de la curva epidémica.

**(4)** El inicio de la vacunación en la tercera curva redujo el número de casos en la curva siguiente.

**(5)** El aislamiento social no cumplido por parte de los jóvenes afecta la dinámica de la pandemia.

## Introducción

 La pandemia causada por el coronavirus SARS-CoV-2 fue considerada una crisis global de salud pública. En enero de 2021, el síndrome respiratorio agudo grave ocasionado por el SARS-CoV-2 resultó en más de 83 millones de casos confirmados y más de 1,8 millones de muertes globalmente ^( 1 )^ . El espectro clínico de la COVID-19 (del inglés *coronavirus disease* 2019) es amplio, abarcando infección asintomática, fiebre, fatiga, mialgias, enfermedad leve del tracto respiratorio superior, neumonía viral grave con riesgo de muerte que requiere hospitalización, y en algunos casos puede ocasionar la muerte ^( 2 )^ . 

 Brasil, en el período de febrero de 2020 a octubre de 2022, registró aproximadamente 34 millones de casos de COVID-19, conformando una tasa de incidencia de 16.567 casos/100 mil habitantes y una tasa de letalidad del 2,0%. Paraná, en el mismo período, registró cerca de 2,8 millones de casos, con una incidencia de 24.076 casos/100 mil habitantes (siendo el tercer estado del país en número de casos) y una tasa de letalidad del 0,6% ^( 3 )^ . A su vez, el municipio de Foz do Iguaçu-PR, notificó 76.839 casos y 1.331 óbitos confirmados por la enfermedad hasta la primera semana de octubre de 2022, correspondiendo a una tasa de incidencia de casos confirmados de 29.786/100 mil habitantes y una tasa de mortalidad de 513/100 mil habitantes ^( 4 )^ . 

 El inicio de la pandemia y su expansión por medio de la transmisión comunitaria corresponde al pico epidémico inicial, diferente del virus de la gripe, el betacoronavirus es de característica no estacional, lo que ha diseminado la enfermedad incluso en verano ^( 5 )^ . En el momento cuando ocurrió el relajamiento de las medidas de aislamiento y de distanciamiento social resurgió el aumento del número de casos y óbitos y la ocurrencia de un nuevo pico, caracterizado por el aumento de casos, dado la falta de vacuna o medicamentos, conforme observado en el período. De este modo, cada país presentó un pico y una curva epidémica y estos ocurrieron en momentos distintos durante los años de 2020 y 2021 ^( 6 )^ . 

 Debido a la extensa franja territorial de fronteras de Brasil, siendo esta de aproximadamente 16.886 kilómetros, divididas con diez países vecinos, que se relacionan por medios turísticos, comerciales, económicos y sanitarios, se tornó imprescindible el acompañamiento de la situación epidemiológica de los casos de COVID-19 y su evolución, y el análisis del impacto de la pandemia en estas regiones ^( 3 )^ . 

 Considerando que el municipio de Foz do Iguaçu-PR, forma parte de la mayor región de tríplice frontera de Brasil, siendo ella entre Brasil, Paraguay y Argentina, caracterizada como la principal frontera de América del Sur en términos de población, circulación diaria de personas oriundas de todos los continentes debido al turismo internacional, relaciones comerciales de compra y venta de bienes de consumo y duraderos y de acceso a los sistemas de salud ^( 7 )^ , se conformó en la ciudad de frontera internacional en el país con mayor incidencia de casos y óbitos por COVID-19, justificando su elección para la realización de este estudio. 

 Además en este espacio el elevado riesgo de transmisión de la COVID-19 estaba potencializado por la alta circulación de personas de diferentes nacionalidades y, en respuesta, se tomaron medidas de cribado y cierre de fronteras terrestres (puestos de control), marítimas (puertos) y aéreas (aeropuertos), aislamiento de grupos de alto riesgo, distanciamiento social, diagnósticos y pruebas masivas ^( 8^ - ^9 )^ . 

 Las variaciones en las manifestaciones clínicas se componen entre las diferencias en la composición etaria, morbilidades, condiciones sociales, así como la discrepancia de cultura, de estructura social y de cuidados de salud entre los países y regiones. Así se tornó fundamental comprender e identificar las principales características clínicas y epidemiológicas de los pacientes con SARS-CoV-2, a fin de reconocer el perfil más vulnerable a la enfermedad ^( 10 )^ . 

 Destaca aún, que los problemas políticos, sanitarios y económicos agravados por la pandemia de la COVID-19 pusieron en relieve la necesidad de estudiar las peculiaridades de esta región fronteriza ^( 7 )^ . El objetivo de este artículo es describir las curvas epidémicas y analizar el perfil epidemiológico de los pacientes hospitalizados por la COVID-19 en un municipio de tríplice frontera. 

## Método

### Diseño del estudio

 Se trata de un estudio descriptivo-cuantitativo, con datos retrospectivos, desarrollado en conformidad con las directrices del *Checklist for Reporting Results of Internet E-Surveys* y *Strengthening the Reporting of Observational Studies in Epidemiology* (STROBE) ^( 11 )^ . 

### Escenario del estudio

 Realizado en el municipio de Foz do Iguaçu-PR, ubicado en la triple frontera entre Brasil, Paraguay y Argentina. Cuenta con una población de 257.971 habitantes ^( 12 )^ . 

 Durante el período de la pandemia, el municipio disponía de 589 camas hospitalarias, siendo el 71% proporcionadas por el Sistema Único de Saúde (SUS). Es destacable que el 97,8% de la población del municipio estaba cubierta por Equipos de Salud Familiar. Dispone de 30 Unidades Básicas de Salud, tres Centros de Atención Psicosocial y tres Unidades de Pronto Atención. Tanto el SUS como los servicios privados del municipio atienden a la población local y regional, así como a visitantes y ciudadanos argentinos y paraguayos que buscan acceso al sistema de salud ^( 13^ - ^14 )^ . 

### Población del estudio

La población involucrada en el estudio configuró la totalidad de los pacientes que fueron notificados y confirmados para COVID-19 en Foz do Iguaçu-PR en el período de marzo de 2020 a diciembre de 2021, obtenidos mediante notificación por el Banco de Datos Notifica COVID-19. Se excluyeron los casos sin información de diagnóstico de COVID-19 por RT-PCR (reacción de transcriptasa reversa seguida de reacción en cadena de la polimerasa) y de confirmación de hospitalización cuando se trataba de los análisis de las curvas epidémicas entre hospitalizados.

### Período

Los datos fueron recopilados entre marzo de 2020 y diciembre de 2021 del Banco Notifica COVID-19, del Departamento de Vigilancia Epidemiológica Municipal.

### Variables bajo estudio y análisis de los datos

Inicialmente se identificaron las curvas epidémicas de acuerdo con las semanas epidemiológicas de COVID-19 obtenidas en el período mencionado, así como la prevalencia por 100.000 habitantes del municipio de Foz do Iguaçu-PR, siendo el número de casos presentado en forma de gráficos de columnas. Para ello se utilizaron las frecuencias semanales de los casos positivos, siendo que el eje horizontal representa el tiempo y el vertical las frecuencias. El mismo gráfico también fue elaborado para el número de internaciones ocurridas en los períodos de las curvas epidémicas.

 La asignación de los períodos de las semanas epidemiológicas, se realizó con base en el concepto epidemiológico de curva epidémica, designado como representación gráfica del número de casos de una determinada enfermedad por fecha de inicio de la misma, siendo que cada formación de valle ocurre una nueva curva epidémica, que proporciona información como la magnitud, tendencia en el tiempo y patrón de propagación de los casos, entre otros ^( 15 )^ . 

Posteriormente, se analizaron los pacientes que presentaron prueba positiva para COVID-19 y fueron sometidos a hospitalización en unidades hospitalarias de Foz do Iguaçu-PR, totalizando 2927 casos. En esta etapa, se realizó la caracterización del perfil de estos pacientes a partir de las siguientes variables cualitativas: sexo, grupo etario, raza/color, país de residencia, municipio de residencia, ocupación, paciente institucionalizado, gestante y evolución. Estas fueron sumarizadas y compiladas en tablas mediante frecuencias absolutas (n) y relativas (%), para cada curva epidémica encontrada. Las frecuencias absolutas de estas variables fueron comparadas entre las curvas mediante la prueba de chi-cuadrado. Cuando los presupuestos para la realización de la prueba de chi-cuadrado no fueron cumplidos, se realizó la prueba exacta de Fisher. Cuando tales pruebas apuntaron a una asociación estadísticamente significativa entre las variables, se realizó aún el análisis de residuos estandarizados y ajustados, de tal forma que residuos por encima de 1,96 indicaron asociación positiva entre las variables y aquellos menores que -1,96 indicaron asociación negativa. Para todas las pruebas, se adoptó un nivel de significancia de 0,05. Tales análisis fueron realizados en el programa XlStat Versión 2014.

### Aspectos éticos

Considerando la Resolución nº 466 del 12 de diciembre de 2012 del Consejo Nacional de Salud y demás directrices y normas reguladoras respecto a las investigaciones involucrando seres humanos, el proyecto de investigación fue aprobado por el Comité de Ética en Investigaciones Involucrando Seres Humanos de la Universidad Estatal del Oeste de Paraná – UNIOESTE bajo el dictamen 4.894.155.

## Resultados

### Identificación de curvas epidémicas según semanas epidemiológicas de mayor prevalencia

 Conforme se muestra en la Figura 1 , al evaluar el período de marzo de 2020 a diciembre de 2021, se obtuvieron 4 curvas epidémicas, las cuales abarcaron las siguientes semanas epidemiológicas: 

Curva Epidémica 1 - ocurrió en el período del 31/05/2020 al 10/10/2020, correspondiente a la 23ª a la 41ª semana epidemiológica y se diagnosticaron 7682 personas con COVID-19.

Curva Epidémica 2 - ocurrió en el período del 11/10/2020 al 02/01/2021, correspondiente a la 42ª a la 53ª semana epidemiológica y se diagnosticaron 9249 personas con COVID-19.

Curva Epidémica 3 - ocurrió en el período del 03/01/2021 al 17/04/2021, correspondiente a la 1ª a la 15ª semana epidemiológica y se diagnosticaron 11478 personas con COVID-19.

Curva Epidémica 4 – ocurrió en el período del 18/04/2021 al 07/08/2021, correspondiente a la 16ª a la 31ª semana epidemiológica y se diagnosticaron 7408 personas con COVID-19.

 Observando las líneas ascendentes del número de casos diagnosticados de COVID-19, se verificaron los picos en las semanas 29 y 48 del año 2020, y semanas 9 y 22 del año 2021 (en el gráfico identificadas como semanas 62 y 75). Las semanas epidemiológicas 8ª a 11ª de 2021 (semanas 61 a 64 del estudio), en la tercera curva epidémica, presentaron las mayores frecuencias absolutas de casos positivos para COVID-19 (n = 1110, 430 casos/100.000 habitantes; n = 1345, 521 casos/100.000 habitantes; n = 1201, 465 casos/100.000 habitantes y n = 914, 354 casos/100.000 habitantes) ( Figura 1 ). 

**Figura 1.** Frecuencia absoluta de casos positivos de COVID-19, por semanas epidemiológicas, en el período de marzo de 2020 a diciembre de 2021 en Foz do Iguaçu, PR, Brasil

### Perfil de los pacientes hospitalizados en cada curva epidémica

 En total, fueron hospitalizados con COVID-19, 504 (6,56% de los diagnosticados) pacientes durante la primera curva epidémica, 520 (5,62% de los diagnosticados) pacientes en la segunda curva epidémica, 988 (8,61% de los diagnosticados) pacientes en la tercera curva epidémica y 915 (12,35% de los diagnosticados) en ocasión de la cuarta curva epidémica. Se verificó que el mayor número de hospitalizaciones ocurrió durante las curvas 3 y 4 de la pandemia ( Figura 2 ). 

 Se pudo verificar que la mayoría de los pacientes hospitalizados con COVID-19 pertenecían al sexo masculino, tenían edades entre 46 y 85 años, raza/color blanco, eran residentes del propio municipio y país, presentaban ocupación no vinculada al área de la seguridad pública y salud y evolucionaron hacia la cura. Una pequeña parte de los casos que necesitaron hospitalización estaba institucionalizada o era gestante ( Tabla 1 ). 

 Entre las variables asociadas a la curva 1, se encontró edad de 0 a 15 años y de 61 a 85 años, raza/color blanco y amarillo, residencia en Foz do Iguaçu y Brasil y casos que curaron. La curva 2 estuvo asociada a edad de 61 a 85 años, residencia en Brasil, estar viviendo en instituciones de residencia prolongada para ancianos y clínicas de recuperación y evolucionar hacia la cura. Asociados a la curva 3, se encontraron personas de 61 a 85 años, de raza/color pardo, residentes en otros municipios que no son Foz do Iguaçu y desenlace muerte. La curva 4 estuvo asociada a personas con edad entre 16 y 60 años, raza/color pardo, residir en otros países y desenlace cura ( Tabla 1 ). 

**Figura 2.** Frecuencia absoluta de las hospitalizaciones por COVID-19, por semanas epidemiológicas, en el período de las curvas epidémicas de 2020 y 2021 en Foz do Iguaçu, PR, Brasil

**Tabla 1.** Frecuencias absolutas (n) y relativas (%) de las variables cualitativas de pacientes hospitalizados con COVID-19, para cada curva epidémica. Foz do Iguaçu, PR, Brasil, 2020-2021

**Variables**	**Categorías**	**Curva 1**	**Curva 2**	**Curva 3**	**Curva 4**	**p-valor** ^†^
		n* (%)	n* (%)	n* (%)	n* (%)	
Sexo	Masculino	306(60,7)	291(56,0)	564(57,1)	559(61,1)	0,128 ^†^
	Femenino	198(39,3)	229(44,0)	424(42,9)	356(38,9)	
Franja etaria	0 a 15 años	17(3,4) ^‡^	10(1,9)	25(2,5)	10(1,1) ^§^	<0,001 ^†^
	16 a 30 años	24(4,8)	29(5,6)	45(4,6)	68(7,4) ^‡^	
	31 a 45 años	80(15,9)	61(11,7) ^§^	148(14,9) ^§^	226(24,7) ^‡^	
	46 a 60 años	133(26,4) ^§^	165(31,7)	296(30,0)	338(36,9) ^‡^	
	61 a 85 años	228(45,2) ^‡^	234(45,0) ^‡^	444(44,9) ^‡^	263(28,7) ^§^	
	86 a 97 años	22(4,4) ^‡^	21(4,0)	30(3,0)	10(1,1) ^§^	
Raza/Color	Blanca	239(57,0) ^‡^	224(51,6)	404(48,7)	382(48,1)	<0,001 ^†^
	Parda	89(21,2) ^§^	192(44,2)	394(47,8) ^‡^	379(47,7) ^‡^	
	Amarilla	77(18,4) ^‡^	6(1,4) ^-^	10(1,2) ^§^	10(1,3) ^§^	
	Negra/Indígena	14(3,3)	12(2,8)	22(2,7)	23(2,9)	
País de residencia	Brasil	500(99,2) ^‡^	517(99,4) ^‡^	962(97,4)	883(96,5) ^§^	<0,001 ^†^
	Otros	4(0,8) ^§^	3(0,6) ^§^	26(2,6)	32(3,5) ^‡^	
Municipio de residencia	Foz do Iguaçu	504(100) ^‡^	511(98,3)	955(96,7) ^‡^	893(97,6)	<0,001 ^†^
	Otros	-	9(1,7)	33(3,3) ^‡^	22(2,4)	
Ocupación	Otros	255(95,5)	352(98,6)	652(99,1)	565(99,8)	- ^||^
	Seguridad pública/Salud	12(4,5)	5(1,4)	6(0,9)	1(0,2)	
Pacientes institucionalizados	Unidades penitenciarias	-	1(33,3) ^§^	27(93,1)	10(83,3)	0,036 ^**^
	IRPA ^¶^ y clínicas de recuperación	-	2(66,7) ^‡^	2(6,9)	2(16,7)	
Embarazada	No	80(97,6)	67(94,4)	209(97,7)	268(98,2)	0,304 ^**^
	Sí	2(2,4)	4(5,6)	5(2,3)	5(1,8)	
Evolución	Cura	311(68,4) ^‡^	277(67,2) ^‡^	383(47,8) ^§^	511(65,6) ^‡^	<0,001 ^†^
	Fallecimientos	144(31,6) ^§^	135(32,8) ^§^	419(52,2) ^‡^	268(34,4) ^§^	

* n = Muestra, difiere ya que datos en blanco, ignorados y no aplicables no fueron considerados en el análisis

† p-valor = Prueba de chi-cuadrado

‡ Residuos estandarizados y ajustados por encima de 1,96 que indicaron asociación positiva entre las categorías de respuesta de las variables

§ Residuos por debajo de -1,96 que indicaron asociación negativa

|| No fue posible aplicar ninguna prueba para esta variable

¶ IRPA – Institución de Residencia Prolongada para Ancianos

** p-valor de la prueba exacta de Fisher

## Discusión

Este estudio busca comprender la ocurrencia de las curvas epidémicas entre los casos hospitalizados por COVID-19. El estudio de las curvas epidémicas es fundamental para prever futuras demandas de hospitalización, la frecuencia del surgimiento de nuevas variantes virales y la efectividad de las intervenciones sanitarias, incluyendo la vacunación.

 La pandemia en Brasil tomó proporciones crecientes entre la primera y segunda curva epidémica. En ese mismo período, Brasil y los Estados Unidos de América ya eran considerados países epicentro de la enfermedad ^( 16 )^ . 

 También hubo un aumento del 161% de casos nuevos notificados entre estos dos períodos ^( 17 )^ , hecho también observado en el estudio sobre el colapso del sistema de salud en Manaus, que registró 56 mil casos en la primera curva y el doble en la segunda ^( 18 )^ . 

 El estado de Paraná evidenció tendencia de aceleración de la transmisión del COVID-19 entre el inicio y mediados del mes de abril de 2021. La 9ª Regional de Salud de Paraná, que involucra a Foz do Iguaçu-PR, se destacó negativamente en ese período, con el mayor coeficiente de incidencia de la enfermedad ^( 19 )^ . 

 Como medida de mitigación de la enfermedad, el municipio de Foz do Iguaçu adoptó las medidas restrictivas dictadas por el estado de Paraná e implementó el Decreto Nº 27.994/20, que contempla las medidas de prevención, buscando el cuidado de la población y manteniendo el sistema de vigilancia en alerta ^( 20 )^ . En la fecha de publicación del decreto, aún no había registro de contagios positivos, ni de casos sospechosos, siendo el primer caso positivo notificado el día 18 de marzo de 2020, clasificado como un caso importado, y el primer fallecimiento por el virus ocurrió el 26 de abril del mismo año. 

 Conforme la Figura 1 , se observa un retraso entre el primer caso confirmado y el inicio de la primera curva, hecho que puede ser justificado por las severas medidas restrictivas implantadas por el municipio y por Paraguay y Argentina. 

 Por otro lado, la primera curva epidémica surge a finales del mes de mayo de 2020, período que coincide con la publicación del Decreto Nº 28.103, que flexibilizó las medidas restrictivas, autorizando la apertura del comercio aunque con límites de aforo. Este Decreto delimitó las medidas restrictivas a barrios de la ciudad con mayor incidencia, que eran establecidas a partir de la evaluación por georreferenciamiento de la morbimortalidad ^( 21 )^ . 

 La ocurrencia de la segunda curva epidémica puede ser resultado de la flexibilización de las medidas protectoras de aislamiento, así como la apertura del Puente Internacional de la Amistad que conecta Brasil con Paraguay y la interrupción de las acciones de control de barreras sanitarias en todos los puntos de acceso a la ciudad ^( 22 )^ . En esa ocasión, Ciudad del Este, en Paraguay, presentaba una incidencia de 1.544 casos por 100 mil habitantes y una tasa de letalidad del 3,9% y Foz do Iguaçu acumulaba una incidencia de 3.065 casos por 100 mil habitantes y una tasa de letalidad del 1,5% ^( 23^ - ^24 )^ . 

 Con la apertura del Puente de la Amistad, se retomaron las relaciones de convivencia familiar y laboral y conforme el registro de la Secretaría de Salud de Foz do Iguaçu, ocurrió un incremento del 30% de pruebas para diagnóstico de COVID-19, correspondiendo a paraguayos y brasileños emigrados al país vecino, que buscaron atención en los servicios de salud del municipio ^( 24 )^ . En este estudio se verificó un aumento del número de no residentes en Brasil hospitalizados en Foz do Iguaçu. 

 Es importante mencionar que, en general, algunas medidas de control fronterizo fueron más efectivas que otras. Sin embargo, mucho permanece incierto sobre este tema ^( 25 )^ . 

 La tercera curva epidémica coincidió con el período de aumento de la aglomeración de personas debido a las festividades de fin de año y carnaval. La tercera ola se mostró la más contagiosa y letal, llegando a 401 casos diarios y un pico de 1.106 casos activos ^( 4 )^ . Lo que puede ser explicado en parte por la circulación de nuevas variantes en Brasil (B.1.1.7, 20B/501Y.V1 o VOC-202012/01) ^( 26 )^ , que también alcanzaron el municipio. Para controlar la situación se publicó un nuevo decreto, el Nº 6983, el cual determinaba la suspensión de las clases presenciales y el servicio y actividades no esenciales en todo el estado y toque de queda de las 20 a las 05 horas. Dicho decreto tuvo vigencia hasta el 08 de marzo de 2021 ^( 7 , 27 )^ . 

 Un estudio con datos de fronteras internacionales en todos los continentes mostró la eficacia de las medidas introducidas para restringir la circulación humana ^( 28 )^ , siendo la cuarentena la respuesta más eficaz frente a la pandemia del coronavirus ^( 25 )^ . 

 La cuarta curva epidémica ocurrió en un período de flexibilización de las medidas restrictivas ^( 25 )^ y la circulación de una nueva variante denominada Beta (B.1.351 o 501Y.V2), que hasta febrero de 2021 había sido detectada en 35 países. La confirmación de registros de esta variante en Brasil ocurrió a finales de abril de 2021 ^( 26 )^ . 

El análisis temporal de los resultados de este estudio, de los decretos de cierre y apertura de las fronteras en Brasil (específicamente la frontera entre Foz do Iguaçu y Ciudad del Este), la flexibilización de las medidas de aislamiento y distanciamiento social, y el surgimiento de nuevas variantes virales llevaron a la percepción de la relación entre estos eventos y el aumento en el número de casos de COVID-19, hospitalizaciones y la ocurrencia de muertes.

El Puente Internacional de la Amistad fue reabierta el 15 de octubre de 2020 durante el feriado de Proclamación de la República, este hecho ocurrió en la 42ª semana epidemiológica y a continuación ocurrieron los demás eventos (citados anteriormente) y a partir de entonces, según la presente investigación, se evidenció también el aumento en el número de casos en las semanas epidemiológicas siguientes (45ª a 48ª) en la ciudad de Foz do Iguaçu.

 Sin embargo, sobre el cierre de fronteras internacionales, hay información que muestra que estas pueden estar entre las medidas de control del COVID-19 más costosas desde el punto de vista económico ^( 25 )^ . Se destaca que las políticas introducidas para restringir la circulación humana dentro de un país fueron más eficaces en respuesta a la pandemia del coronavirus que el cierre de las fronteras internacionales ^( 28 )^ . 

 En cuanto a las curvas epidémicas, se destaca el aumento creciente de la incidencia, hospitalización y muerte por COVID-19 en el período de la tercera curva epidémica en el municipio, durante los primeros cuatro meses del año 2021, esta ocurrencia fue concordante con el escenario de agravamiento de la pandemia en el contexto internacional. En este período, solo el 13,7% de la población brasileña había sido vacunada contra el SARS-CoV-2 ^( 27 )^ . 

 Factores como el curso de la enfermedad, el surgimiento de nuevas variantes con mayor transmisibilidad, las aglomeraciones clandestinas y las aglomeraciones de fin de año, se sumaron al crecimiento de la curva, cuando todavía solo había medidas no farmacológicas para la mitigación de la infección por COVID-19 ^( 29 )^ . 

 El perfil epidemiológico mostró mayor ocurrencia de hospitalizaciones entre hombres de raza blanca. Al considerar los grupos etarios según las curvas, se notó mayor frecuencia entre los hombres hospitalizados de 61 a 85 años, en la tercera curva epidémica, aspectos concordantes con otro estudio ^( 30 )^ . 

El análisis de los casos hospitalizados por grupo etario según las curvas epidémicas confirma la alta circulación/transmisión y número de enfermos por SARS-CoV2 en la localidad, ya que el grupo etario de 0 a 15, 61 a 85 y 86 y más años de edad, obtuvo mayor porcentaje de hospitalización durante la tercera curva epidémica (x2 = 132,09; p < 0,0001).

 Para los pacientes en el grupo de edad de 61 y más años, hallazgos científicos corroboran con la presente investigación, visto que las personas mayores que normalmente poseen comorbilidades tales como hipertensión arterial sistémica, cardiopatías, diabetes mellitus, enfermedades respiratorias crónicas e inmunosupresoras forman parte de un grupo de riesgo que generalmente cursa con hospitalización y mal pronóstico ^( 31 )^ . 

 Por otro lado, la hospitalización y la mortalidad por COVID-19 en pacientes hospitalizados mostraron una reducción, especialmente entre las personas mayores en la proporción de aproximadamente un tercio en la cuarta curva epidémica. La existencia de vacuna anticipadamente y mayor adhesión a la segunda dosis podría haber evitado un número significativo de muertes entre ancianos en el período de enero a abril de 2021 ^( 32 )^ . 

 En cuanto a las características de los hospitalizados según las curvas epidémicas, este estudio fue concordante con hallazgos en Sudáfrica, al comparar el número de curvas, (cuatro) períodos de ocurrencia y edad de los pacientes durante la cuarta curva epidémica, y diverge en cuanto a la presencia de un mayor número de mujeres en la tercera curva epidémica ^( 30 )^ . 

 Este estudio apoya el descubrimiento de que los hombres son más propensos a ser infectados y que necesitan más frecuentemente hospitalización por COVID-19 ^( 30 , 32 )^ , potencialmente debido a diferencias inmunológicas basadas en el sexo, variaciones de género o comorbilidades asociadas, incluyendo hipertensión, enfermedad cardiovascular, enfermedad pulmonar, patrones y prevalencia de fumar y consumo de alcohol ^( 33 )^ . Asimismo, otros hallazgos revelaron que el 69% de todas las admisiones hospitalarias eran de sexo masculino, representando el 74% de todas las admisiones en unidad de cuidados intensivos y el 77% de toda la mortalidad por COVID-19 ^( 34^ - ^35 )^ . 

 En Brasil, pequeños cambios ocurrieron en la proporción de personas mayores y blancos infectados en la segunda curva epidémica en comparación con la primera, también se observó un leve aumento en la hospitalización en la segunda curva epidémica ^( 18 )^ . Sin embargo, se observó un número creciente de hospitalizaciones durante 2020 y 2021 en Foz do Iguaçu - PR, lo cual no coincidió exactamente con el mismo período en otros escenarios nacionales, dado que Brasil es un país continental, heterogéneo, con diversas densidades poblacionales y grandes inequidades regionales, que determinan tiempos distintos para el inicio y el fin de cada curva epidémica ^( 36 )^ . 

 El cambio en el perfil de los infectados con un aumento de la contribución de individuos de raza/color parda en Foz do Iguaçu (a partir de la segunda curva) puede haber sido reflejo de las desigualdades en el acceso a los servicios de salud entre blancos, pardos y negros. Aunque el SARS-CoV-2 afecta a la población en general, sus impactos pueden ser diferentes debido a la desigualdad socioeconómica, al racismo estructural y a situaciones de exclusión social que forman parte de la realidad brasileña. Aquellos con condiciones de vida y trabajo más precarios tienen mayores dificultades en el acceso a bienes y servicios esenciales, lo que puede resultar en diferencias observables por variables *proxy de estatus* socioeconómico como la raza/color ^( 7 )^ . 

 En cuanto al lugar de residencia, los enfermos hospitalizados en Foz do Iguaçu provenientes de otras localidades del estado de Paraná y del exterior, ocurrió debido a la regionalización del SUS (ya que por la Central de Camas los pacientes son trasladados de sus municipios a las ciudades con camas disponibles), por la hospitalización de extranjeros en viaje de turismo y negocios y por el acceso de enfermos provenientes de Paraguay y Argentina. Además, a partir de la apertura del Puente Internacional de la Amistad, aumentó el número de hospitalizaciones por COVID-19 de brasileños y extranjeros originarios de Paraguay ^( 24 )^ . 

 En el estado de Paraná hubo un aumento de casos en noviembre de 2020, en la 46ª semana epidemiológica, donde la tasa media de ocupaciones hospitalarias era del 89% en el estado y del 96% en la capital, Curitiba ^( 37 )^ . En cuanto a la frontera, el aumento de casos posiblemente se relacionó con el feriado prolongado del día 15 de noviembre, fecha en que la ciudad de Foz do Iguaçu superó las expectativas de visitaciones desde agosto de 2020, con una ocupación del 95% de los hoteles de la ciudad ^( 25 )^ . 

 En este estudio, fue poco frecuente la hospitalización de profesionales de la salud durante las cuatro curvas epidémicas, a diferencia de lo observado en un estudio realizado en Alemania y Malasia, que solo en los primeros cinco meses del año 2020 contabilizaron el 8,9% del total de notificaciones clasificadas como de profesionales de salud enfermos por COVID-19 que necesitaron ser hospitalizados ^( 38 )^ . 

 En cuanto a las personas institucionalizadas, se observó que durante la tercera curva epidémica aumentó el número de personas privadas de libertad con complicaciones por COVID-19 que necesitaron hospitalización. El análisis de la categoría de pacientes institucionalizados, respecto a la necesidad de hospitalización debido al COVID-19, demostró que el grupo de residentes en instituciones penitenciarias fueron más vulnerables en comparación con las personas fuera de las prisiones ^( 39 )^ . 

 El registro de gestantes hospitalizadas por COVID-19 en este estudio fue bajo, incluso durante la tercera curva epidémica. Se sabe que las gestantes son más propensas a la hospitalización en unidad de cuidados intensivos, requieren ventilación invasiva, necesitan oxigenación por membrana extracorpórea y evolucionan hacia el óbito con mayor frecuencia que las mujeres no embarazadas en edad reproductiva ^( 40 )^ . 

 Destaca que el período con mayor morbilidad y mortalidad por COVID-19 registrado en el municipio fue durante la tercera curva epidémica, que corresponde al período de enero a abril de 2021, presentando 419 muertes entre aquellos que fueron hospitalizados. Según los datos consolidados por el Panel Coronavirus del Ministerio de Salud, cuando Brasil registraba la segunda curva epidémica, la frontera Brasil-Paraguay-Argentina estaba viviendo el momento más crítico de la tercera curva epidémica, con un altísimo porcentaje de hospitalizaciones y muertes por la enfermedad ^( 18 )^ . 

 La vacunación contra el SARS-CoV-2 en el municipio comenzó siguiendo las orientaciones de la 9ª Regional de Salud de Paraná y los profesionales de salud que estaban en la línea de frente, seguidos de los ancianos alojados en instituciones asilares, fueron los primeros en recibir la primera dosis de la vacuna y en este período la triple frontera ya experimentaba los impactos de la tercera curva epidémica, que tuvo su pico el 14 de febrero de 2021, registrando más de 1800 casos nuevos en la 8ª semana epidemiológica de 2021 ^( 27 )^ . 

 En Brasil, con el inicio del verano, las infecciones entre los jóvenes aumentaron considerablemente, marcando así la segunda curva epidémica. Tanto en Brasil como en otros países, a diferencia del primer momento de la pandemia, los casos positivos crecieron entre los jóvenes en este período. Tal ocurrencia puede justificarse por el hecho de que gran parte de esta población cree ser menos susceptible a la contaminación por COVID-19, por no formar parte de grupos de riesgo con predisposición para la enfermedad ^( 41 )^ . 

 El verano, período característico de vacaciones y los feriados ocurridos en la tercera curva epidémica, son otros factores que pueden haber contribuido a la explosión de la incidencia de casos y hospitalizaciones en Foz do Iguaçu. Según datos del Parque Nacional de Iguaçu, el número de turistas en las Cataratas de Iguaçu en enero de 2021 fue de 75 mil turistas, el mayor número registrado desde el inicio de la pandemia ^( 25 )^ . 

 Destaca que en períodos de feriados, en que las personas viajan y promueven reuniones, consecuentemente culminan en aglomeraciones y, estas contribuyeron a la diseminación de la COVID-19 ^( 42 )^ . La propagación del virus a escala global fue favorecida por la circulación de personas y objetos infectados. Sin embargo, el proceso salud-enfermedad en una región fronteriza posee características particulares asociadas a la movilidad de personas entre países diariamente ^( 43 )^ . 

La observación de las curvas epidémicas de COVID-19 en el municipio de Foz do Iguaçu muestra que las medidas sanitarias elegidas se asociaron con la morbilidad y mortalidad de la enfermedad. Visto que, la implementación de las medidas restrictivas antes del registro de la primera curva epidémica, tuvo impacto sobre la incidencia de la enfermedad y la hospitalización y cuando el municipio adoptó medidas de flexibilización hubo un aumento en el número de casos; posteriormente, las medidas restrictivas fueron intensificadas logrando un decrecimiento en las curvas, tras algunas semanas.

 Sobre este hecho, en el contexto de un resurgimiento del SARS-CoV-2, una estrategia de control mediante la prohibición de eventos públicos y reuniones públicas de más de diez personas mostró asociación con la reducción de casos en un 6% al 7º día, 13% al 14º día y 29% al 28º día ^( 44 )^ . 

 Las acciones de restricción de ajustes públicos y la cobertura vacunal en Brasil y en los municipios fronterizos, que había superado el 70% de cobertura de la primera dosis y, en el municipio de Foz do Iguaçu, siendo del 92,5% (primera dosis) y del 86,3% para la segunda dosis ^( 24 )^ , impactaron en la reducción de hospitalizaciones en la cuarta curva epidémica. 

 En este estudio, la frecuencia de profesionales de salud hospitalizados fue pequeña, observándose mayor frecuencia durante la primera curva epidémica. A pesar de ello, las repercusiones psicosociales y físicas durante la pandemia en la salud, especialmente de los profesionales de enfermería, fueron severas, dado el tiempo de exposición, ritmo y complejidad en el trabajo frente a las personas enfermas por COVID-19 ^( 45 )^ . 

Cabe resaltar la existencia de incompletitud en algunas categorías de respuesta en la base de datos utilizada en el estudio, esta situación pudo haber limitado la interpretación de algunas variables. Sin embargo, para mitigar sesgos se analizaron 35.817 registros de casos positivos para COVID-19 en el período e, incluyó solo las notificaciones con información completa sobre las variables dependientes e independientes. Además, los casos considerados en el estudio obtuvieron confirmación de diagnóstico para SARS-CoV-2 por RT-PCR y estado de hospitalización.

La identificación del perfil de los pacientes hospitalizados por COVID-19 en cada curva epidémica, contribuye a mostrar la importancia del análisis de datos de vigilancia epidemiológica para el planeamiento de medidas sanitarias. Fue posible identificar que, a pesar de que las personas de raza/color parda conformaban el 30% del total poblacional, aún así, obtuvieron un patrón de enfermedad y muerte similar a las personas de raza/color blanca, (63,3% del total de la población); también se verificó que hombres y personas privadas de libertad como grupos vulnerables, siendo estos potenciales marcadores de riesgo para hospitalización y muerte por COVID-19. Otra contribución para el manejo de futuras epidemias es la necesidad de disponibilidad de vacunas en tiempo oportuno, visto que en este caso, podría haber prevenido el exceso de enfermedad y muertes, como se identificó durante la tercera curva epidémica de la enfermedad en esta localidad de frontera internacional.

## Conclusión

El análisis epidemiológico de los casos de COVID-19 en el municipio de Foz do Iguaçu que necesitaron hospitalización, permitió concluir que las curvas epidémicas fueron influenciadas por diferentes situaciones. Durante la primera curva prácticamente toda la población no tenía inmunidad específica, sumado el agravante de la presencia de grupos de riesgo y la flexibilización de las medidas restrictivas con apertura del comercio. Precediendo a la segunda curva, marcó la reapertura del Puente Internacional de la Amistad e interrupción de las acciones de control de barreras sanitarias. La tercera curva epidémica fue observada en un período tras el aumento de la aglomeración de personas debido a las festividades de fin de año, carnaval y circulación de nuevas variantes en Brasil denominadas B.1.1.7, 20B/501Y.V1 y VOC-202012/01. A su vez, el aumento creciente de la incidencia, hospitalización y muerte por COVID-19, se configuró en una cuarta curva epidémica un espacio de tiempo después del surgimiento de otra variante del SARS-CoV-2 denominada Beta (B.1.351 o 501Y.V2).

En cuanto al perfil de los enfermos hospitalizados, se concluyó que en el período de mayor transmisibilidad de la enfermedad (tercera curva epidémica), ser del sexo masculino, personas de raza/color parda, estar en el rango de edad de 61 a 85 años y vivir en una unidad prisional se asociaron con la hospitalización y ocurrencia de muerte.

# Translation: Curvas epidêmicas e o perfil de pacientes hospitalizados pela COVID-19 em região de fronteira

**DOI:** 10.1590/1518-8345.6772.4297

**Published:** 2024-09-23

**Authors:** Merielly Kunkel, Érica Alves Ferreira Gordillo, Laiz Mangini Cicchelero, Franz Porzsolt, Mara Cristina Ripoli Meira, Helder Ferreira, Neide Martins Moreira, Larissa Djanilda Parra da Luz, Nathalia Halax Orfão, Reinaldo Antonio Silva-Sobrinho

**Affiliations:** 1 Universidade Estadual do Oeste do Paraná, Centro de Educação Letras e Saúde, Foz do Iguaçu, PR, Brasil.; 2 Institute of Clinical Economics, Alemanha.; 3 Secretaria de Saúde de Foz do Iguaçu, Vigilância Epidemiológica, Foz do Iguaçu, PR, Brasil.; 4 Universidade Federal de Rondônia, Porto Velho, RO, Brasil.

**Keywords:** COVID-19, Controle de Doenças Transmissíveis, Hospitalização, Saúde Pública, Saúde na Fronteira, Pandemias

## Abstract

**Objetivo:**

descrever as curvas epidêmicas e analisar o perfil epidemiológico dos pacientes hospitalizados pela COVID-19 em um município de tríplice fronteira.

**Método:**

descritivo-quantitativo. A população configurou-se de casos de COVID-19 que necessitaram de hospitalização, analisando variáveis como: idade, sexo, raça/cor, município de residência, ocupação, gestante, paciente institucionalizado e evolução. Utilizou-se a análise estatística descritiva e os testes análise de variância e qui-quadrado.

**Resultados:**

identificaram-se quatro curvas epidêmicas no período estudado. Dentre os casos hospitalizados, predominou o sexo masculino (55%). A cura foi o desfecho mais frequente nas curvas 1, 2 e 4, porém sem diferença estatística (p = 0,2916). A curva 3 apresentou uma maior frequência de óbitos (41,70%) em relação à cura (38,77%). As médias de idade foram significativamente diferentes entre as curvas, sendo que a curva 4 apresentou a menor média de idade.

**Conclusão:**

concluiu-se que as curvas epidêmicas foram influenciadas por diferentes situações; população não vacinada, flexibilização das medidas restritivas, reabertura da fronteira Brasil-Paraguai, interrupção das ações de controle, aglomeração de pessoas e circulação de novas variantes. Por meio do perfil epidemiológico dos doentes hospitalizados, concluiu-se que ser do sexo masculino, de raça/cor parda, na faixa etária de 61 a 85 anos, estar privado de liberdade se associaram com a hospitalização e ocorrência de óbito.

## Destaques

**(1)** A terceira curva epidêmica, em abril de 2021, apresentou a maior frequência de óbitos.

**(2)** Homens com idade média de 56 anos foram o público mais vulnerável para a doença.

**(3)** O fechamento e abertura da fronteira impactou no comportamento da curva epidêmica.

**(4)** O início da vacinação na terceira curva reduziu o número de casos na curva seguinte.

**(5)** O isolamento social não cumprido por parte dos jovens afeta a dinâmica da pandemia.

## Introdução

 A pandemia causada pelo coronavírus SARS-CoV-2 foi considerada uma crise global de saúde pública. Em janeiro de 2021, a síndrome respiratória aguda grave ocasionada pelo SARS-CoV-2 resultou em mais de 83 milhões de casos confirmados e mais de 1,8 milhões de mortes globalmente ^( 1 )^ . O espectro clínico da COVID-19 (do inglês *coronavirus disease* 2019) é amplo, abrangendo infecção assintomática, febre, fadiga, mialgias, doença leve do trato respiratório superior, pneumonia viral grave com risco de morte que exige internação hospitalar e, em alguns casos, pode ocasionar a morte ^( 2 )^ . 

 No período de fevereiro de 2020 a outubro de 2022, o Brasil registrou aproximadamente 34 milhões de casos de COVID-19, conformando taxa de incidência de 16.567 casos/100 mil habitantes e taxa de letalidade de 2,0%. O Paraná, no mesmo período, registrou cerca de 2,8 milhões de casos, com uma incidência de 24.076 casos/100 mil habitantes (sendo o terceiro estado do país em número de casos) e taxa de letalidade de 0,6% ^( 3 )^ . Por sua vez, o município de Foz de Iguaçu-PR, notificou 76.839 casos e 1.331 óbitos confirmados pela doença até a primeira semana de outubro de 2022, correspondendo a uma taxa de incidência de casos confirmados 29.786/100 mil habitantes e uma taxa de mortalidade de 513/100 mil habitantes ^( 4 )^ . 

 O início da pandemia e sua expansão por meio da transmissão comunitária corresponde ao pico epidêmico inicial. Diferente do vírus da gripe, o betacoronavírus é de característica não sazonal, o que tem disseminado a doença até mesmo no verão ^( 5 )^ . No momento quando ocorreu o relaxamento das medidas de isolamento e de distanciamento social ressurgiu o aumento do número de casos, de óbitos e a ocorrência de novo pico, tendo em vista a falta de vacina ou medicamentos, conforme observado no período. Desse modo, cada país apresentou um pico e uma curva epidêmica os quais ocorreram em momentos distintos durante os anos de 2020 e 2021 ^( 6 )^ . 

 Devido à extensa faixa territorial de fronteiras do Brasil – aproximadamente 16.886 quilômetros – divididas com dez países vizinhos que se relacionam por meios turísticos, comerciais, econômicos e sanitários, tornou-se imprescindível o acompanhamento da situação epidemiológica dos casos da COVID-19 e sua evolução, tanto quanto a análise do impacto da pandemia nessas regiões ^( 3 )^ . 

 Considerando que o município de Foz do Iguaçu-PR faz parte da maior região de tríplice fronteira do Brasil com o Paraguai e a Argentina, conformou-se a referida cidade de contatos internacionais como aquela com maior incidência de casos e óbitos por COVID-19, justificando sua escolha para realização deste estudo. Caracterizada como a principal fronteira da América do Sul em termos de população e circulação diária de pessoas oriundas de todos os continentes, sobretudo devido ao turismo internacional, às relações comerciais de compra e venda de bens de consumo e duráveis e de acesso aos sistemas de saúde ^( 7 )^ . 

 Ademais, nesse espaço, o elevado risco de transmissão da COVID-19 estava potencializado pela alta circulação de pessoas de pessoas de diferentes nacionalidades e, em resposta, foram tomadas medidas de triagem e fechamento de fronteiras terrestres (postos de controle), marítimas (portos) e aéreas (aeroportos), isolamento de grupos de alto risco, distanciamento social, diagnósticos e testagem em massa ^( 8^ - ^9 )^ . 

 As variações nas manifestações clínicas compõem-se entre as diferenças na composição etária, morbidades, condições sociais, como também a discrepância de cultura, de estrutura social e de cuidados de saúde entre os países e regiões. Assim se tornou fundamental compreender e identificar as principais características clínicas e epidemiológicas dos pacientes com SARS-CoV-2, a fim de reconhecer o perfil mais vulnerável à doença ^( 10 )^ . 

 Destaca-se, ainda, que os problemas políticos, sanitários e econômicos agravados pela pandemia da COVID-19 colocaram em relevo a necessidade de estudar as peculiaridades dessa região fronteiriça ^( 7 )^ . Sendo assim, o objetivo deste artigo é descrever as curvas epidêmicas e analisar o perfil epidemiológico dos pacientes hospitalizados pela COVID-19 em um município de tríplice fronteira. 

## Método

### Delineamento do estudo

 Trata-se de um estudo descritivo-quantitativo, com dados retrospectivos, desenvolvido em conformidade com as diretrizes do *Checklist for Reporting Results of Internet E-Surveys guidelines and Strengthening the Reporting of Observational Studies in Epidemiology* (STROBE) ^( 11 )^ . 

### Cenário do estudo

 Realizado no município de Foz do Iguaçu - PR, localizado na tríplice fronteira Brasil-Paraguai-Argentina. Possui população de 257.971 habitantes ^( 12 )^ . 

 No período da pandemia, o município disponibilizava 589 leitos hospitalares, sendo 71% disponibilizados pelo Sistema Único de Saúde (SUS). Destaca-se que 97,8% da população do município era coberta por Equipes de Saúde da Família. Possui 30 Unidades Básicas de Saúde, três Centros de Atenção Psicossocial e três Unidades de Pronto Atendimento. Tanto o SUS quanto os serviços privados do município atendem à população local e regional, bem como visitantes e cidadãos Argentinos e Paraguaios que buscam acesso ao sistema de saúde ^( 13^ - ^14 )^ . 

### População do estudo

A população envolvida no estudo configurou a totalidade dos pacientes que foram notificados e confirmados para COVID-19 em Foz do Iguaçu-PR no período de março de 2020 a dezembro de 2021, obtidos por meio de notificação pelo Banco de Dados Notifica COVID-19. Foram excluídos os casos sem informação de diagnóstico de COVID-19 por RT-PCR (reação de transcriptase reversa seguida de reação em cadeia da polimerase) e de confirmação de hospitalização quando se tratou das análises das curvas epidêmicas entre hospitalizados.

### Período

Os dados foram coletados entre o período de março de 2020 a dezembro de 2021 do Banco Notifica COVID-19, do Departamento de Vigilância Epidemiológica Municipal.

### Variáveis sob estudo e análise dos dados

Inicialmente, identificou-se as curvas epidêmicas de acordo com as semanas epidemiológicas da COVID-19, obtidas no período supracitado, com a prevalência por 100.000 habitantes do município de Foz do Iguaçu-PR, sendo o número de casos apresentado em forma de gráficos de colunas. Para tal, foram utilizadas as frequências semanais dos casos positivos, sendo que o eixo horizontal representa o tempo e o vertical as frequências. O mesmo gráfico também foi elaborado para o número de internações ocorridas nos períodos das curvas epidêmicas.

 A alocação dos períodos das semanas epidemiológicas foram realizadas com base no conceito epidemiológico de curva epidêmica, designado como a representação gráfica do número de casos de determinada doença pela data de início da mesma, sendo que a cada formação de vale ocorre uma nova curva epidêmica, a qual fornece informações como a magnitude, tendência no tempo e padrão de propagação dos casos, entre outras ^( 15 )^ . 

Posteriormente, analisou-se os pacientes que apresentaram teste positivo para a COVID-19 e foram submetidos à internação em unidades hospitalares de Foz do Iguaçu-PR, totalizando 2927 casos. Nessa etapa, foi realizada a caracterização do perfil dos pacientes a partir das seguintes variáveis qualitativas: sexo, faixa etária, raça/cor, país de residência, município de residência, ocupação, paciente institucionalizado, gestante e evolução. Estas foram sumarizadas e compiladas em tabelas por meio de frequências absolutas (n) e relativas (%), para cada curva epidêmica encontrada. As frequências absolutas destas variáveis foram comparadas entre as curvas por meio do teste de qui-quadrado. Quando os pressupostos para a realização do teste qui-quadrado não foram atendidos, realizou-se o teste exato de Fisher. Quando tais testes apontaram para uma associação estatisticamente significante entre as variáveis, fez-se, ainda, uma análise de resíduos padronizada e ajustada, de tal forma que resíduos acima de 1,96 indicaram associação positiva entre as variáveis e aqueles menores que -1,96 indicaram associação negativa. Para todos os testes, adotou-se um nível de significância de 0,05. Tais análises foram realizadas no programa XlStat Versão 2014.

### Aspectos éticos

Considerando a Resolução nº 466 de 12 de dezembro de 2012 do Conselho Nacional de Saúde e demais diretrizes e normas regulamentadoras quanto às pesquisas envolvendo seres humanos, o projeto de pesquisa foi aprovado pelo Comitê de Ética em Pesquisas Envolvendo Seres Humanos da Universidade Estadual do Oeste do Paraná – UNIOESTE sob o parecer 4.894.155.

## Resultados

### Identificação de curvas epidêmicas segundo semanas epidemiológicas de maior prevalência

 Conforme disposto na Figura 1 , ao avaliar o período de março de 2020 a dezembro de 2021, foram obtidas 4 curvas epidêmicas, as quais englobaram as seguintes semanas epidemiológicas: 

Curva Epidêmica 1 - ocorreu no período de 31/05/2020 a 10/10/2020, correspondente a 23ª à 41ª semana epidemiológica e foram diagnosticadas 7682 pessoas com COVID-19.

Curva Epidêmica 2 - ocorreu no período de 11/10/2020 a 02/01/2021, correspondente a 42ª à 53ª semana epidemiológica e foram diagnosticadas 9249 pessoas com COVID-19.

Curva Epidêmica 3 - ocorreu no período de 03/01/2021 a 17/04/2021, correspondente a 1ª à 15ª semana epidemiológica e foram diagnosticadas 11478 pessoas com COVID-19.

Curva Epidêmica 4 – ocorreu no período de 18/04/2021 a 07/08/2021, correspondente a 16ª à 31ª semana epidemiológica e foram diagnosticadas 7408 pessoas com COVID-19.

 Observando as linhas ascendentes de número de casos diagnosticados da COVID-19, verificou-se picos nas semanas 29 e 48 do ano de 2020, e semanas 9 e 22 do ano de 2021 (no gráfico identificadas como semanas 62 e 75). As semanas epidemiológicas de 8ª a 11ª de 2021 (semanas 61 a 64 do estudo), na terceira curva epidêmica, apresentaram as maiores frequências absolutas de casos positivos para COVID-19 (n = 1110, 430 casos/100.000 habitantes; n = 1345, 521 casos/100.000 habitantes; n = 1201, 465 casos/100.000 habitantes e n = 914, 354 casos/100.000 habitantes) ( Figura 1 ). 

**Figura 1.** Frequência absoluta de casos positivos da COVID-19, por semanas epidemiológicas, no período de março de 2020 a dezembro de 2021 em Foz do Iguaçu, PR, Brasil

### Perfil dos pacientes hospitalizados em cada curva epidêmica

 Ao total, foram hospitalizados 504 (6,56% dos diagnosticados) pacientes com COVID-19 durante a primeira curva epidêmica; 520 (5,62% dos diagnosticados) pacientes na segunda curva epidêmica; 988 (8,61% dos diagnosticados) pacientes na terceira curva epidêmica e 915 (12,35% dos diagnosticados) por ocasião da quarta curva epidêmica. Verificou-se que o maior número de internações ocorreu durante as curvas 3 e 4 da pandemia ( Figura 2 ). 

 Foi possível verificar que a maioria dos pacientes hospitalizados com COVID-19 pertencia ao sexo masculino, possuía idade entre 46 e 85 anos, sendo da raça/cor branca, eram residentes do próprio município e país, apresentavam ocupação não vinculada à área da segurança pública e saúde e evoluíram para cura. Uma pequena parcela dos casos que necessitaram internação estava institucionalizada ou era gestante ( Tabela 1 ). 

 Dentre as variáveis associadas à curva 1, encontrou-se idade de 0 a 15 anos e de 61 a 85 anos, raça/cor branca e amarela, residência em Foz do Iguaçu, Brasil, sendo casos que curaram. A curva 2 esteve associada à idade de 61 a 85 anos, com residência no Brasil, morando em instituições de longa permanência para idosos e clínicas de recuperação e evoluindo para cura. Associados à curva 3, foram encontradas pessoas de 61 a 85 anos, de raça/cor parda, residentes em outros municípios que não Foz do Iguaçu e desfecho óbito. A curva 4 esteve associada a pessoas com idade entre 16 e 60 anos, raça/cor parda, residir em outros países e desfecho cura ( Tabela 1 ). 

**Figura 2.** Frequência absoluta das hospitalizações por COVID-19, por semanas epidemiológicas, no período das curvas epidêmicas de 2020 e 2021 em Foz do Iguaçu, PR, Brasil

**Tabela 1.** Frequências absolutas (n) e relativas (%) das variáveis qualitativas de pacientes hospitalizados com COVID-19, para cada curva epidêmica. Foz do Iguaçu, PR, Brasil, 2020-2021

**Variáveis**	**Categorias**	**Curva 1**	**Curva 2**	**Curva 3**	**Curva 4**	**p-valor** ^†^
		n*(%)	n*(%)	n*(%)	n*(%)	
Sexo	Masculino	306(60,7)	291(56,0)	564(57,1)	559(61,1)	0,128 ^†^
	Feminino	198(39,3)	229(44,0)	424(42,9)	356(38,9)	
Faixa etária	0 a 15 anos	17(3,4) ^‡^	10(1,9)	25(2,5)	10(1,1) ^§^	<0,001 ^†^
	16 a 30 anos	24(4,8)	29(5,6)	45(4,6)	68(7,4) ^‡^	
	31 a 45 anos	80(15,9)	61(11,7) ^§^	148(14,9) ^§^	226(24,7) ^‡^	
	46 a 60 anos	133(26,4) ^§^	165(31,7)	296(30,0)	338(36,9) ^‡^	
	61 a 85 anos	228(45,2) ^‡^	234(45,0) ^‡^	444(44,9) ^‡^	263(28,7) ^§^	
	86 a 97 anos	22(4,4) ^‡^	21(4,0)	30(3,0)	10(1,1) ^§^	
Raça/Cor	Branca	239(57,0) ^‡^	224(51,6)	404(48,7)	382(48,1)	<0,001 ^†^
	Parda	89(21,2) ^§^	192(44,2)	394(47,8) ^‡^	379(47,7) ^‡^	
	Amarela	77(18,4) ^‡^	6(1,4) ^-^	10(1,2) ^§^	10(1,3) ^§^	
	Preta/Indígena	14(3,3)	12(2,8)	22(2,7)	23(2,9)	
País de residência	Brasil	500(99,2) ^‡^	517(99,4) ^‡^	962(97,4)	883(96,5) ^§^	<0,001 ^†^
	Outros	4(0,8) ^§^	3(0,6) ^§^	26(2,6)	32(3,5) ^‡^	
Município de residência	Foz do Iguaçu	504(100) ^‡^	511(98,3)	955(96,7) ^‡^	893(97,6)	<0,001 ^†^
	Outros	-	9(1,7)	33(3,3) ^‡^	22(2,4)	
Ocupação	Outros	255(95,5)	352(98,6)	652(99,1)	565(99,8)	- ^||^
	Segurança pública/Saúde	12(4,5)	5(1,4)	6(0,9)	1(0,2)	
Pacientes institucionalizados	Unidades prisionais	-	1(33,3) ^§^	27(93,1)	10(83,3)	0,036 ^**^
	ILPI ^¶^ e clínicas de recuperação	-	2(66,7) ^‡^	2(6,9)	2(16,7)	
Gestante	Não	80(97,6)	67(94,4)	209(97,7)	268(98,2)	0,304 ^**^
	Sim	2(2,4)	4(5,6)	5(2,3)	5(1,8)	
Evolução	Cura	311(68,4) ^‡^	277(67,2) ^‡^	383(47,8) ^§^	511(65,6) ^‡^	<0,001 ^†^
	Óbitos	144(31,6) ^§^	135(32,8) ^§^	419(52,2) ^‡^	268(34,4) ^§^	

* n = Amostra, difere uma vez que dados em branco, ignorados e não se aplica não foram considerados na análise

† p-valor =Teste de qui-quadrado

‡ Resíduos padronizados e ajustados acima de 1,96 que indicaram associação positiva entre as categorias de resposta das variáveis

§ Resíduos abaixo de -1,96 que indicaram associação negativa

|| Não foi possível aplicar nenhum teste para esta variável

¶ ILPI = Instituição de longa permanência para idosos

** p-valor do teste exato de Fisher

## Discussão

Este estudo busca compreender a ocorrência das curvas epidêmicas entre os casos hospitalizados por COVID-19. O estudo das curvas epidêmicas é fundamental para prever futuras demandas de hospitalização, frequência do surgimento de novas variantes virais e efetividade das intervenções sanitárias, entre elas a vacinação.

 A pandemia no Brasil tomou proporções crescentes entre a primeira e segunda curva epidêmica. Nesse mesmo período, o Brasil, e os Estados Unidos da América já eram considerados países epicentro da doença ^( 16 )^ . 

 Também houve um aumento de 161% de casos novos notificados entre esses dois períodos ^( 17 )^ , fato também observado no estudo sobre o colapso do sistema de saúde em Manaus, que registrou 56 mil casos na primeira curva e o dobro na segunda ^( 18 )^ . 

 O estado do Paraná evidenciou uma tendência de aceleração da transmissão da COVID-19 entre o início e metade do mês de abril de 2021. A 9ª Regional de Saúde do Paraná, que envolve Foz do Iguaçu-PR, destacou-se negativamente nesse período, com maior coeficiente de incidência da doença ^( 19 )^ . 

 Como medida de mitigação da doença, o município de Foz do Iguaçu adotou as medidas restritivas decretadas pelo estado do Paraná e implementou o Decreto Nº 27.994/20, contemplando as medidas de prevenção, visando o cuidado da população e mantendo o sistema de vigilância em alerta ^( 20 )^ . Na data de publicação do decreto, ainda não havia registro de contágios positivos nem de casos suspeitos, sendo que o primeiro caso positivo foi notificado no dia 18 de março de 2020, classificado como um caso importado, e o primeiro óbito pelo vírus ocorreu no dia 26 de abril do mesmo ano. 

 Conforme a Figura 1 , observa-se um retardo entre o primeiro caso confirmado e o início da primeira curva, fato que pode ser justificado pelas severas medidas restritivas implantadas pelo município e pelo Paraguai e Argentina. 

 Por outro lado, a primeira curva epidêmica surge no final do mês de maio de 2020, período coincidente com a publicação do Decreto N ^o^ 28.103, que flexibilizou as medidas restritivas, autorizando a abertura do comércio embora houvesse limites de frequentadores. Esse Decreto delimitou as medidas restritivas a bairros da cidade com maior incidência, que eram estabelecidas a partir da avaliação por georreferenciamento da morbimortalidade ^( 21 )^ . 

 A ocorrência da segunda curva epidêmica pode ser resultado da flexibilização das medidas protetivas de isolamento, da abertura da Ponte Internacional da Amizade que liga o Brasil ao Paraguai, bem como da interrupção das ações controle de barreiras sanitárias em todos os locais de acesso à cidade ^( 22 )^ . Nessa ocasião, a Ciudad del Leste, no Paraguai, apresentava incidência de 1.544 casos por 100 mil habitantes e taxa de letalidade de 3,9% enquanto Foz do Iguaçu acumulava incidência de 3.065 casos por 100 mil habitantes e taxa de letalidade de 1,5% ^( 23^ - ^24 )^ . 

 Com a abertura da Ponte da Amizade, as relações de convívio familiar e de trabalho foram retomadas. Conforme o registro da Secretaria de Saúde de Foz do Iguaçu, ocorreu um incremento de 30% de teste para diagnóstico da COVID-19, correspondendo aos paraguaios e brasileiros imigrados para o país vizinho que buscaram atendimento nos serviços de saúde do município ^( 24 )^ . Neste estudo, verificou-se o aumento do número de não residentes no Brasil internamentos em Foz do Iguaçu. Torna-se importante mencionar que, no geral, algumas medidas de controle fronteiriço foram mais eficazes do que outras, entretanto, muito permanece incerto sobre esse tema ^( 25 )^ . 

 A terceira curva epidêmica coincidiu com o período de aumento da aglomeração de pessoas devidos às festividades de final de ano e carnaval. A terceira onda se mostrou a mais contagiosa e letal, chegando a 401 casos diários e um pico de 1.106 casos ativos ^( 4 )^ , o que pode ser explicado, em parte, pela circulação de novas variantes no Brasil (B.1.1.7, 20B/501Y.V1 ou VOC-202012/01) ^( 26 )^ , que alcançaram também o município. Para controle da situação foi publicado um novo decreto, o de Nº 6983, o qual determinava a suspensão das aulas presenciais e o serviço e atividades não essenciais em todo estado, além do toque de recolher estabelecido das 20 às 05 horas. Tal decreto teve vigência até 08 de março de 2021 ^( 7 , 27 )^ . 

 Um estudo com dados de fronteiras internacionais em todos os continentes mostrou a eficácia das medidas introduzidas para restringir a circulação humana ^( 28 )^ , sendo a quarentena a resposta mais eficaz frente à pandemia do coronavírus ^( 25 )^ . 

 A quarta curva epidêmica ocorreu em um período de flexibilização das medidas restritivas ^( 25 )^ e de circulação de uma nova variante denominada Beta (B.1.351 ou 501Y.V2), que, até fevereiro de 2021, fora detectada em 35 países. A confirmação de registros dessa variante no Brasil ocorreu no final de abril de 2021 ^( 26 )^ . 

A análise temporal dos resultados deste estudo, dos decretos de fechamento e da abertura das fronteiras no Brasil (especificamente a fronteira entre Foz do Iguaçu e Ciudad del Leste), a flexibilização das medidas de isolamento, distanciamento social e o surgimento de novas variantes virais levaram à percepção da relação entre esses eventos, ao aumento no número de casos de COVID-19, de hospitalizações e à ocorrência de óbitos.

A Ponte Internacional da Amizade foi reaberta em 15 de outubro de 2020 no feriado de Proclamação da República, esse fato ocorreu na 42ª semana epidemiológica. Em seguida, ocorreram os demais eventos (citados anteriormente) e, a partir de então, de acordo com a presente pesquisa, foi evidenciado também o aumento no número de casos nas semanas epidemiológicas seguintes (45ª à 48ª), na cidade de Foz do Iguaçu.

 Entretanto, sobre o fechamento de fronteiras internacionais, há informações que mostram que estas podem estar entre as medidas de controle da COVID-19 mais dispendiosas do ponto de vista econômico ^( 25 )^ . Destaca-se que as políticas introduzidas para restringir a circulação humana dentro de um país foram mais eficazes quanto à resposta à pandemia do coronavírus do que o fechamento das fronteiras internacionais ^( 28 )^ . 

 Em relação às curvas epidêmicas, destaca-se o aumento crescente da incidência, da hospitalização e de óbitos pela COVID-19 no período da terceira curva epidêmica no município, durante os quatro primeiros meses do ano de 2021. Tal ocorrência estava de acordo com o cenário de agravamento da pandemia no contexto internacional. Neste período, apenas 13,7% da população brasileira tinha sido vacinada contra o SARS-CoV-2 ^( 27 )^ . 

 Fatores como o curso da doença, o surgimento de novas variantes com maior transmissibilidade, as aglomerações clandestinas e aglomerações no fim de ano somaram-se ao crescimento da curva, quando ainda só havia medidas não farmacológicas para a mitigação da infecção por COVID-19 ^( 29 )^ . 

 O perfil epidemiológico mostrou maior ocorrência de internações entre homens da raça/cor branca. Ao considerar as faixas etárias segundo as curvas, notou-se maior frequência entre os homens hospitalizados com idade de 61 a 85 anos, na terceira curva epidêmica, aspectos concordantes com outro estudo ^( 30 )^ . 

 A análise dos casos hospitalizados por faixa etária segundo as curvas epidêmicas confirma a elevada circulação/transmissão e o número de doentes pelo SARS-CoV2 na localidade, visto que a faixa etária de 0 a 15, de 61 a 85 e 86 e mais anos de idade, obtiveram maior percentual de hospitalização durante a terceira curva epidêmica (x ^2^ = 132,09; p < 0,0001). 

 Para os doentes na faixa de idade de 61 e mais anos, os achados científicos corroboram com a presente pesquisa, visto que pessoas idosas que normalmente possuem comorbidades tais como hipertensão arterial sistêmica, cardiopatias, diabetes mellitus, doenças respiratórias crônicas e imunossupressoras fazem parte de um grupo de risco, geralmente cursando com hospitalização e mau prognóstico ^( 31 )^ . 

 Por outro lado, a hospitalização e a mortalidade pela COVID-19 em pacientes hospitalizados apresentaram redução, especialmente entre idosos na proporção de aproximadamente um terço na quarta curva epidêmica. A existência de vacina antecipadamente e a maior adesão à segunda dose poderiam ter evitado um expressivo número de óbitos entre idosos no período de janeiro a abril de 2021 ^( 32 )^ . 

 Em se tratando das características dos hospitalizados segundo as curvas epidêmicas, este estudo foi concordante com achados na África do Sul, ao comparar o número de curvas, (quatro) períodos de ocorrência e idade dos pacientes durante a quarta curva epidêmica, divergindo quanto à presença de maior número de mulheres na terceira curva epidêmica ^( 30 )^ . 

 Este estudo apoia a descoberta de que os homens são mais propensos a serem infectados e que necessitam mais frequentemente de hospitalização por COVID-19 ^( 30 , 32 )^ , potencialmente devido a diferenças imunológicas baseadas no sexo, variações de gênero ou comorbidades associadas, incluindo hipertensão, doença cardiovascular, doença pulmonar, padrões e prevalência de tabagismo e consumo de álcool ^( 33 )^ . Do mesmo modo, outros achados revelaram que 69% de todas as admissões em hospitais eram do sexo masculino, representando 74% de todas as admissões em unidade de terapia intensiva e 77% de toda a mortalidade pela COVID-19 ^( 34^ - ^35 )^ . 

 No Brasil, pequenas mudanças ocorreram na proporção de idosos e brancos infectados na segunda curva epidêmica em comparação com a primeira, observou-se também leve aumento de hospitalização na segunda curva epidêmica ^( 18 )^ . Contudo, foi observado um crescente número de hospitalizações no decorrer de 2020 e 2021 em Foz do Iguaçu - PR, não coincidindo exatamente com o mesmo período em outros cenários nacionais, visto que o Brasil é um país continental, heterogêneo, com variadas densidades populacionais e grandes iniquidades regionais, que determinam tempos distintos para o início e término de cada curva epidêmica ^( 36 )^ . 

 A mudança do perfil dos infectados com um aumento da contribuição de indivíduos de raça/cor parda em Foz do Iguaçu (a partir da segunda curva) pode ter sido reflexo das desigualdades no acesso aos serviços de saúde entre brancos, pardos e negros. Embora o SARS-CoV-2 afete a população como um todo, seus impactos podem ser diferentes por conta da desigualdade socioeconômica, devido ao racismo estrutural e a situações de exclusão social que fazem parte da realidade brasileira. Aqueles que possuem condições de vida e trabalho mais precários têm maiores dificuldades no acesso aos bens e serviços essenciais, podendo, então, resultar em diferenças observáveis por variáveis proxy de status socioeconômico como é o caso de raça/cor ^( 7 )^ . 

 Em relação ao local de residência, o cenário de doentes hospitalizados em Foz do Iguaçu oriundos de outras localidades do estado do Paraná e do exterior se deu devido a regionalização do Sistema Único de Saúde (visto que pela Central de Leitos os pacientes são deslocados de seus municípios para as cidades com leitos disponíveis), pela hospitalização de estrangeiros em viagens de turismo e negócios e pelo acesso de doentes oriundos do Paraguai e Argentina. Ademais, a partir da abertura da Ponte Internacional da Amizade, aumentou-se o número de hospitalizações por COVID-19 de brasileiros e estrangeiros oriundos do Paraguai ^( 24 )^ . 

 No estado do Paraná houve aumento dos casos em novembro de 2020, na 46ª semana epidemiológica, onde a taxa média de ocupações hospitalares era de 89% no estado, e de 96% na capital Curitiba ^( 37 )^ . Em se tratando da fronteira, o aumento de casos possivelmente se relacionou com o feriado prolongado do dia 15 de novembro, data em que cidade de Foz do Iguaçu superou as expectativas de visitações desde agosto de 2020, com uma lotação de 95% dos hotéis da cidade ^( 25 )^ . 

 Neste estudo, foi pouco frequente a hospitalização de profissionais de saúde, durante as quatro curvas epidêmicas, ao contrário do observado em um trabalho realizado na Alemanha e Malásia, no qual somente nos primeiros cinco meses do ano de 2020 contabilizaram-se 8,9% do total de notificações classificadas como de profissionais de saúde doentes por COVID-19 que precisaram ser hospitalizados ^( 38 )^ . 

 Em se tratando de pessoas institucionalizadas, observou-se que durante a terceira curva epidêmica, aumentou-se o número de pessoas privadas de liberdade com complicações por COVID-19 e que necessitaram de hospitalização. A análise da categoria pacientes institucionalizados quanto à necessidade de hospitalização devido à COVID-19 demostrou que os grupos de moradores em instituições prisionais foram mais vulneráveis quando comparados às pessoas fora das prisões ^( 39 )^ . 

 Foi baixo o registro de gestantes hospitalizadas por COVID-19 neste estudo, mesmo durante a terceira curva epidêmica. Sabe-se que as gestantes são mais propensas a hospitalização em unidade de terapia intensiva, que requererem ventilação invasiva, que necessitam de oxigenação por membrana extracorpórea e evoluem para óbito mais frequentemente do que mulheres não grávidas em idade reprodutiva ^( 40 )^ . 

 Destaca-se que o período com maior morbimortalidade por COVID-19 registrado no município foi na terceira curva epidêmica, que correspondeu ao período de janeiro a abril de 2021, apresentando 419 óbitos, entre aqueles que foram hospitalizados. Conforme os dados consolidados pelo Painel Coronavírus – Ministério da Saúde, quando o Brasil registrava a segunda curva epidêmica, a fronteira Brasil-Paraguai-Argentina estava vivenciando o momento mais crítico da terceira curva epidêmica, com elevadíssimo percentual de hospitalizações e óbitos pela doença ^( 18 )^ . 

 A vacinação contra o SARS-CoV-2 no município iniciou seguindo as orientações da 9ª Regional de Saúde do Paraná, e os profissionais da área da saúde que estavam na linha de frente, seguido dos idosos abrigados nas instituições asilares foram os primeiros a receber a primeira dose da vacina. Neste período a tríplice fronteira já experimentava os impactos da terceira curva epidêmica, que teve seu ápice em 14 de fevereiro de 2021, registrando mais de 1800 casos novos na 8ª semana epidemiológica de 2021 ^( 27 )^ . 

 No Brasil, com o início do verão, as infecções entre os jovens aumentaram consideravelmente, marcando, assim, a segunda curva epidêmica. Tanto no Brasil como em outros países, diferente do primeiro momento da pandemia, os casos positivos cresceram entre os jovens nesse período. Tal ocorrência pode ser justificada pelo fato de grande parte dessa população acreditar ser menos susceptível à contaminação por COVID-19, por não fazerem parte de grupos de risco em que há predisposição para doença ^( 41 )^ . 

 O verão, período característico de férias e os feriados ocorridos na terceira curva epidêmica, representaram outro fator que pode ter contribuído para a explosão da incidência de casos e hospitalizações em Foz do Iguaçu. Segundo dados do Parque Nacional do Iguaçu, o número de turistas nas Cataratas do Iguaçu em janeiro de 2021 foi de 75 mil turistas, o maior número registrado desde o início da pandemia ^( 25 )^ . 

 Destaca-se que períodos de feriados, em que pessoas viajam e promovem reuniões, consequentemente, culminam em aglomerações que contribuíram para a disseminação da COVID-19 ^( 42 )^ . A propagação do vírus em escala global foi favorecida pela circulação de pessoas e objetos infectados. Contudo, o processo saúde-doença em uma região de fronteira possui características particulares associadas à mobilidade de pessoas entre países diariamente ^( 43 )^ . 

A observação das curvas epidêmicas de COVID-19 no município de Foz do Iguaçu mostra que as medidas sanitárias escolhidas se associaram com a morbimortalidade da doença. Tendo em vista que a implantação das medidas restritivas antes do registro da primeira curva epidêmica provocou impacto sobre a incidência da doença e da hospitalização, quando o município adotou medidas de flexibilização, houve aumento no número de casos; posteriormente, medidas restritivas foram intensificadas logrando um decréscimo nas curvas, após algumas semanas.

 Sobre esse fato, no contexto de um ressurgimento do SARS-CoV-2, uma estratégia de controle por meio da proibição de eventos públicos e reuniões públicas de mais de dez pessoas mostrou associação com a redução de casos na ordem de 6% no 7º dia, 13% no 14º dia e 29% no 28º dia ^( 44 )^ . 

 As ações de restrição de ajustamentos públicos e a cobertura vacinal no Brasil e nos municípios fronteiriços ultrapassou 70% da cobertura vacinal da 1ª dose. Já no município de Foz do Iguaçu, obteve-se 92,5% na primeira dose e 86,3% para segunda dose ^( 24 )^ , o que impactou na redução de hospitalizações na quarta curva epidêmica. 

 Neste estudo, a frequência de profissionais de saúde hospitalizados foi pequena. observou-se maior ocorrência durante a primeira curva epidêmica. Apesar disso, as repercussões psicossociais e físicas durante a pandemia na saúde, especialmente dos profissionais da enfermagem foram severas, considerando o tempo de exposição, ritmo e complexidade no trabalho frente as pessoas adoecidas por COVID-19 ^( 45 )^ . 

Cabe ressaltar a existência de incompletude em algumas categorias de resposta no banco de dados utilizado no estudo. Esta situação pode ter limitado a interpretação de algumas variáveis. Entretanto, para mitigar vieses, analisou-se 35.817 registros de casos positivos para COVID-19 no período, incluindo-se somente as notificações com informações completas sobre as variáveis dependentes e independentes. Ademais, os casos considerados no estudo obtiveram confirmação de diagnóstico para SARS-CoV-2 por RT-PCR e estado de hospitalização.

A identificação do perfil dos pacientes hospitalizados pela COVID-19 em cada curva epidêmica contribui para mostrar a importância da análise de dados de vigilância epidemiológica visando o planejamento de medidas sanitárias. Foi possível identificar que apesar das pessoas da raça/cor parda conformarem 30% do total populacional, ainda assim, elas obtiveram um padrão de adoecimento e óbito similar às pessoas da raça/cor branca, (63,3% do total da população). Verificou-se também homens e pessoas privadas de liberdade como grupos vulneráveis, sendo os potenciais marcadores de risco para hospitalização e óbito por COVID-19. Outra contribuição para manejo de futuras epidemias é a necessidade de disponibilização de vacinas em tempo oportuno, visto que, neste caso, a distribuição poderia ter prevenido o excesso de adoecimento e óbitos, como se identificou durante a terceira curva epidêmica da doença nesta localidade de fronteira internacional.

## Conclusão

A análise epidemiológica dos casos de COVID-19 que necessitaram de hospitalização permitiu concluir que as curvas epidêmicas foram influenciadas por diferentes situações, no município de Foz do Iguaçu. Durante a primeira curva, praticamente toda a população não tinha imunidade específica, e adicionou-se, ainda, o agravante da presença de grupos de risco e a flexibilização das medidas restritivas com abertura do comércio. Antecedendo a segunda curva, marcou-se a reabertura da Ponte Internacional da Amizade e interrupção das ações controle de barreiras sanitárias. A terceira curva epidêmica foi observada em um período após o aumento da aglomeração de pessoas devidos às festividades de final de ano, do carnaval além da circulação de novas variantes no Brasil denominadas B.1.1.7, 20B/501Y.V1 e VOC-202012/01. Por sua vez, o aumento crescente da incidência, hospitalização e óbito pela COVID-19 configurou-se em uma quarta curva epidêmica num espaço de tempo depois do surgimento de outra variante do SARS-CoV-2 denominada Beta (B.1.351 ou 501Y.V2).

Quanto ao perfil dos doentes hospitalizados, concluiu-se que, no período de maior transmissibilidade da doença (terceira curva epidêmica), ser do sexo masculino, pertencer ao grupo de pessoas da raça/cor parda, estar na faixa etária de 61 a 85 anos e viver em unidade prisional se associaram com a hospitalização e ocorrência de óbito.
